# Maternal Western diet programs persistent transcriptomic signatures in offspring PBMCs that are largely reversible by interventions during lactation

**DOI:** 10.3389/fnut.2026.1892621

**Published:** 2026-07-22

**Authors:** Catalina A. Pomar, Ana Valle, Antoni Caimari, Andreu Palou, Mariona Palou, Catalina Picó

**Affiliations:** 1 Nutrigenomics, Biomarkers and Risk Evaluation (NuBE) Group, University of the Balearic Islands (UIB), Mallorca, Spain; 2 Health Research Institute of the Balearic Islands (IdISBa), Palma, Spain; 3 CIBER of Physiopathology of Obesity and Nutrition (CIBEROBN). Instituto de Salud Carlos III (ISCIII), Madrid, Spain; 4 Artificial Intelligence Research Institute of the Balearic Islands (IAIB), Palma, Spain; 5 Eurecat, Centre Tecnològic de Catalunya, Biotechnology Area, Reus, Spain

**Keywords:** leptin, maternal obesity, metabolic programming, obesogenic diets, transcriptomic

## Abstract

Maternal consumption of a Western diet (WD) during critical developmental periods has been shown to program long-term metabolic dysfunction in offspring. However, it remains unclear whether these early exposures leave a persistent transcriptomic imprint into adulthood and whether this signature can be reshaped by postnatal interventions, limiting insight into underlying mechanisms and opportunities for early intervention. Here, we aimed to identify long-term transcriptomic signatures associated with maternal WD exposure during gestation and lactation using peripheral blood mononuclear cells (PBMCs) from adult male and female offspring. We further evaluated the potential of two early-life interventions - maternal dietary normalization and pup leptin supplementation during lactation - to reverse these alterations. The offspring from dams fed a normal-fat diet during gestation and lactation was used as a control group. Considering all groups, the expression of 90 genes differed between the control and WD offspring. Both interventions normalized the expression of most genes altered by maternal WD exposure (83 and 76 out of 90, respectively), shifting the global transcriptomic profile toward control levels. Multivariate analyses identified the 20 highest-ranked contributors to the PLS-DA model distinguishing offspring of WD-fed dams from controls, with stronger discriminatory capacity in males than in females, suggesting coordinated dysregulation of immunometabolic pathways, primarily involving immune and inflammatory signaling, cell fate regulation, and metabolic homeostasis. In conclusion, interventions during lactation may effectively reverse most transcriptomic alterations associated with adverse maternal conditions, supporting this period as a critical window for postnatal reprogramming. These findings suggest that early nutritional interventions may help mitigate long-term health risks associated with adverse maternal diet and support the use of PBMC transcriptomic signatures as potential minimally invasive biomarkers of developmental programming.

## 1 Introduction

The developmental origins of health and disease (DOHaD) concept proposes that early life exposures can have lasting impacts on health and disease risk later in life (1, 2). In fact, maternal obesity and/or consumption of a high-fat diet during pregnancy and lactation may increase the risk of adverse outcomes in the offspring, including obesity and increased risk for cardiometabolic disorders (3–7).

Different lines of preclinical evidence strongly suggest that certain treatments and nutritional strategies can counteract early-life metabolic malprogramming. In this regard, our group previously demonstrated that, in diet-induced obese rats, a nutritional intervention carried out during lactation based on the implementation of a healthy diet can significantly mitigate several detrimental programming effects associated with maternal obesity (7–9). In addition to maternal diet, leptin, a hormone naturally present in milk, also plays a key role in shaping the adult phenotype by improving metabolic health and preventing the development of obesity and related disorders later in life (10–12). Leptin supplementation during the suckling period in rats has been shown to reverse alterations induced by adverse maternal conditions, such as gestational calorie restriction (11, 13). It has also been reported to counteract alterations associated with maternal diet-induced obesity; however, these programming effects appear to depend on maternal diet during lactation, as the metabolic benefits are less evident in the offspring of dams fed an obesogenic diet (7).

Despite the encouraging effects observed with both interventions, maternal dietary improvement during lactation and/or pup leptin supplementation, the molecular pathways and processes underlying the long-term effects of early-life nutritional interventions remain incompletely understood. Elucidating these alterations and identifying accessible biomarkers that reflect persistent changes is crucial for improving biological understanding and potential clinical translation. In this context, peripheral blood mononuclear cells (PBMCs) have emerged as a promising model for transcriptomic profiling, given their non-invasive accessibility and their capacity to reflect pathological states (14, 15). Interestingly, PBMCs act as “sentinel” cells, exhibiting the potential to reflect gene expression profiles of internal tissues in response to environmental changes in the body, making them an effective source for monitoring molecular alterations in distant tissues (14). Their value as a source of circulating biomarkers has been demonstrated in a range of diseases (15), and we and others have previously demonstrated the utility of the PBMC transcriptome in nutrition and obesity research (14, 16–18).

Therefore, this study aimed to evaluate the impact of maternal obesogenic conditions on the offspring transcriptome in adulthood and to determine whether two postnatal interventions, maternal dietary normalization during lactation and pup leptin supplementation during suckling, can mitigate these effects despite a standard diet after weaning. This transcriptomic approach is expected to provide insight into the long-term consequences of early nutritional insults, clarify underlying mechanisms, and identify PBMC-based transcriptomic signatures as potential biomarkers of adult metabolic health.

## 2 Materials and methods

### 2.1 Animal protocol and experimental design

All animal procedures were approved by the Bioethics Committee of the University of the Balearic Islands (Protocol No. 2018/13/AEXP, approved on January 23, 2019), in accordance with institutional guidelines for the care and use of laboratory animals.

We studied a subcohort of rats from a previously described and phenotypically characterized experimental study (7, 9). For the present analysis, only selected experimental groups and a subset of male and female animals in each group were included and subjected to the transcriptomic analyses (*n* = 6 in each group). A schematic overview of the experimental design of the present study is shown in Supplementary Figure 1. In brief, virgin female Wistar rats were housed under controlled conditions (22 °C, 12-h light–dark cycle) with ad libitum access to food and water. Animals were randomly assigned to receive either a standard diet (SD; 3.3 kcal·g^−1^, with 8.4% calories from fat, 72.4% from carbohydrates, and 19.3% from protein; Safe, Augy, France) or a high-fat and high-sucrose diet (WD, Western diet, Research Diets, New Brunswick, United States; 4.7 kcal·g^−1^, with 40.0% calories from fat, 43.0% from carbohydrates, and 17.0% from proteins). The dietary intervention began 1 month before mating.

Pregnant females were housed individually and maintained on their respective diets throughout gestation. On postnatal day 1 (PND1), litters were standardized to 10 pups per dam (preferably five males and five females). During lactation, SD-fed dams remained on the same diet (referred to as C-dams, *n* = 8). WD-fed animals were divided into two groups: one continued on the WD (WD-dams, *n* = 9), and the other was switched to the SD (Rev-dams, *n* = 10).

From PND1 to PND20, the offspring of C-, WD-, and Rev-dams (designated as O-C, O-WD, and O-Rev, respectively) were orally supplemented with either leptin (PeproTech, London, United Kingdom) at a dose equivalent to five times the amount typically ingested from breast milk (12), or vehicle (water).

After weaning at PND21, offspring were maintained on the SD until euthanasia at 4 months of age. Body weight and food intake were monitored throughout the study. Two weeks before sacrifice (at approximately 3.5 months of age), blood samples were collected in heparinized tubes from the saphenous vein without anesthesia under 12 h fasting conditions. Animals were euthanized by decapitation in the fed state. Trunk blood was collected for isolation of peripheral blood mononuclear cells (PBMCs). PBMCs were collected using EDTA (final concentration of 3–4 mM) as anticoagulant and diluted with an equal volume of isosmotic buffered saline. PBMCs were then isolated by OptiPrep density-gradient centrifugation (Sigma Aldrich Química SA, Madrid, Spain) following the manufacturer’s protocol.

### 2.2 RNA extraction

Total RNA was extracted from PBMC using the NucleoSpin TriPrep (Macherey-Nagel, Madrid, Spain), following the manufacturer’s instructions. RNA quantity was measured using a NanoDrop ND-1000 spectrophotometer (NanoDrop Technologies, Inc., Wilmington, DE, United States), and integrity was assessed by 1% agarose gel electrophoresis.

### 2.3 RNA-seq library preparation, sequencing, and analysis

RNA sequencing was conducted on a subset of representative samples from each group (n = 6, a total of 48 samples). Aliquots of total RNA of selected samples were sent to Novogene (Novogene Sequencing Europe, Cambridge, United Kingdom) for whole messenger RNA (mRNA) sequencing. Libraries were prepared using the Novogene NGS RNA Library Prep Set (PT042). mRNA was isolated from total RNA using poly-T oligo-attached magnetic beads, then fragmented and reverse-transcribed into cDNA. Libraries were sequenced using Illumina NovaSeq platforms (19).

Raw reads in fastq format were processed using in-house perl scripts to generate high-quality clean reads by removing adapters, reads containing poly-N, and low-quality reads from the raw data. Quality metrics (Q20, Q30, and GC content) were calculated. All downstream analyses were performed using high-quality clean data.

Clean reads were aligned to the reference genome using Hisat2 v2.0.5. The genome index was built and paired-end reads were mapped using the same software (20). Read counts per gene were obtained using FeatureCounts v1.5.0-p3 (21).

### 2.4 Differential expression analysis and enrichment analysis of differentially expressed genes

Differential expression analysis was restricted to coding transcripts expressed in more than 50% of the animals or in at least *n* = 3 animals in a specific group. Principal Component Analysis (PCA) was performed using MetaboAnalyst (22) for outlier detection. Four samples were excluded accordingly.

Differentially expressed genes (DEGs) between the O-WD and O-C groups were identified using the DESeq2 R package (v1.20.0) (23). DESeq2 provides statistical routines for determining differential expression in digital gene expression data using a model based on the negative binomial distribution. Genes with a *p*-value <0.05 were considered significantly differentially expressed. *p*-values were adjusted using the Benjamini-Hochberg method to control for false discovery rate (FDR). DEGs were visualized using volcano plots generated with the R-package ‘gplots’.

KEGG pathway enrichment analysis was performed using the *clusterProfiler* R package. To reduce bias associated with genome-wide annotations, the background (universe) was restricted to the set of genes detected in the RNA-seq dataset after preprocessing and filtering steps.

### 2.5 Statistical analysis

Statistical analyses related to transcriptomic profiling and pathway enrichment are described above. To identify key transcripts distinguishing between both dietary groups, Partial Least Squares Discriminant Analysis (PLS-DA) was performed comparing O-WD and O-C animals. Model quality was assessed using *R*^2^ and *Q*^2^ parameters derived from cross-validation. This PLS-DA and VIP-based feature selection were conducted using pooled samples from males and females to increase statistical power, given the limited sample size per group, and to prioritize the identification of shared biomarkers across sexes. Differential gene expression between O-WD and O-C animals was assessed using the DESeq2 framework, which is widely used for pairwise comparison in RNA-seq data.

To evaluate the effects of leptin supplementation or maternal diet improvement during lactation on gene expression in offspring, statistical comparisons among O-C, O-WD, O-WD-LEP, and O-REV groups were performed using GraphPad Prism 10.4.1 (GraphPad Software, Boston, MA, United States). To assess overall expression differences across all experimental conditions, a multi-group comparison approach was applied. Data were analyzed both in pooled samples and stratified by sex. Group comparisons were conducted using the Kruskal-Wallis test followed by Dunn’s post-hoc test. Statistical significance was set at *p* ≤ 0.05. Graphs were also generated using GraphPad Prism 10.4.1.

## 3 Results

### 3.1 Phenotypic traits of offspring in adulthood

Phenotypic traits and metabolic parameters of the entire cohort at 4 months of age have been previously reported (7, 8). The phenotypic characteristics of the subcohort included in the present study are shown in Table 1. O-WD females showed significantly higher body weight compared to controls, an effect that was normalized in the O-WD-LEP group and, more markedly, in the O-REV group. No significant differences were observed in adiposity or circulating parameters related to glucose homeostasis and insulin sensitivity. Circulating lipid parameters measured under ad libitum conditions, including total cholesterol and triglycerides, were also comparable across all experimental groups, indicating the absence of systemic lipid alterations in the subcohort.

**Table 1:** Phenotypic and metabolic characteristics of male and female offspring at 4 months of age.

Characteristic	Males				Statistics Kruskal–Wallis	Females				Statistics Kruskal–Wallis
	O-C	O-WD	O-WD-LEP	O-REV		O-C	O-WD	O-WD-LEP	O-REV	
Body Weight (g)	435 ± 11	452 ± 14	471 ± 23	422 ± 11	ns	240 ± 5	261 ± 7	254 ± 6	234 ± 8	**A-B-AB-A**
Fat mass (%)	13.2 ± 1.1	12.2 ± 1.2	13.1 ± 1.4	13.5 ± 0.8	ns	12.7 ± 1.8	13.9 ± 1.3	11.1 ± 1.1	13.1 ± 1.5	ns
iWAT weight (g)	5.58 ± 1.21	6.24 ± 0.42	6.52 ± 0.29	6.19 ± 0.19	ns	2.75 ± 0.15	3.08 ± 0.22	2.53 ± 0.14	2.16 ± 0.24	ns
rpWAT weight (g)	8.66 ± 0.85	10.16 ± 1.21	10.16 ± 0.90	9.90 ± 0.70	ns	2.85 ± 0.14	3.55 ± 0.39	2.58 ± 0.20	2.83 ± 0.40	ns
Glucose Ad libitum (mg/dL)	135 ± 5	143 ± 8	142 ± 9	137 ± 7	ns	139 ± 6	143 ± 9	136 ± 6	124 ± 3	ns
Glucose Fasting (mg/dL)	106 ± 3	101 ± 5	109 ± 7	102 ± 6	ns	83 ± 1	82 ± 4	90 ± 5	86 ± 3	ns
Insulin Fasting (g/L)	0.55 ± 0.10	0.53 ± 0.22	0.61 ± 0.17	0.83 ± 0.14	ns	0.28 ± 0.06	0.29 ± 0.04	0.25 ± 0.02	0.22 ± 0.02	ns
HOMA-IR	3.37 ± 0.59	3.08 ± 1.22	3.59 ± 0.89	5.18 ± 1.00	ns	1.39 ± 0.30	1.45 ± 0.24	1.32 ± 0.11	1.10 ± 0.11	ns
Cholesterol Ad libitum (mg/dL)	108.54 ± 6.75	91.49 ± 16.50	77.23 ± 8.38	92.20 ± 8.31	ns	74.08 ± 10.00	91.69 ± 7.57	106.50 ± 18.94	127.22 ± 14.34	ns
TG Ad libitum (mg/mL)	0.94 ± 0.17	1.26 ± 0.29	1.01 ± 0.16	0.96 ± 0.13	ns	0.40 ± 0.12	0.76 ± 0.16	0.67 ± 0.12	0.42 ± 0.10	ns

Body weight, total body fat percentage, inguinal white adipose tissue (iWAT) weight, retroperitoneal white adipose tissue (rpWAT) weight, ad libitum blood glucose, triglyceride and cholesterol levels, as well as fasting blood glucose, insulin, and HOMA-IR are shown for each experimental group. Data are expressed as mean ± SEM. Experimental groups: O-C, offspring of control dams receiving vehicle; O-WD, offspring of Western diet-fed dams receiving vehicle; O-WD-LEP, offspring of Western diet-fed dams receiving neonatal leptin supplementation; O-REV, offspring of reversion dams receiving vehicle. Group differences were assessed using the Kruskal–Wallis test followed by post-hoc analysis; *p* < 0.05 was considered statistically significant. Different letters indicate statistically significant differences between groups within the same sex; ns, not significant.

### 3.2 Transcriptomic signatures in adult offspring of mothers fed a Western diet

To explore the long-term transcriptomic effects of maternal dietary patterns during gestation and lactation, we first assessed the potential influence of sex on global gene expression profiles. No clear sex-based separation was observed in the PCA or hierarchical clustering based on the transcriptomic data (Supplementary Figure 2), within the resolution of these exploratory analyses. Therefore, male and female offspring were analyzed jointly to increase statistical power and to facilitate the identification of shared gene expression signatures across sexes.

**Figure 1:**
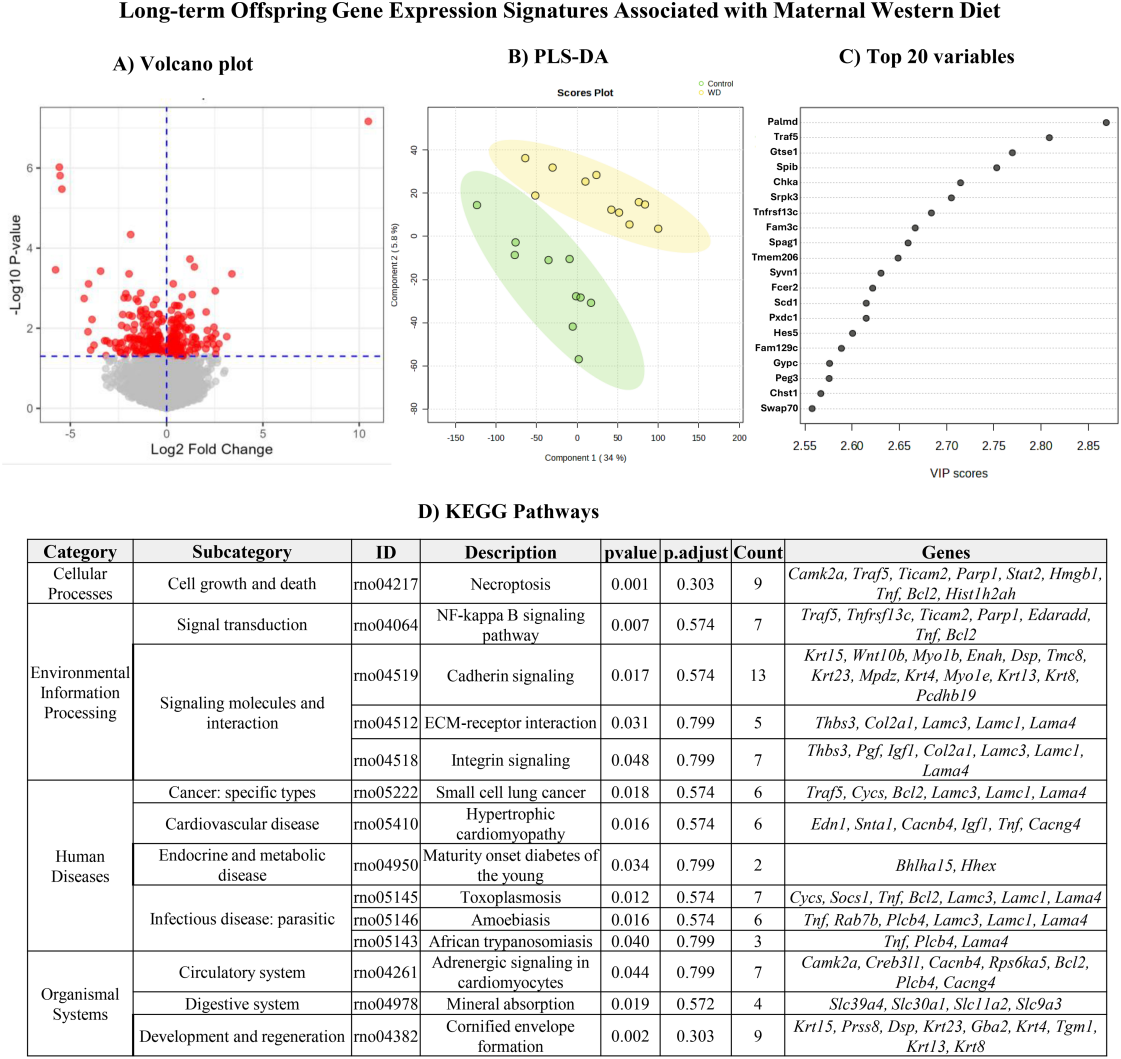
Long-term offspring gene expression signatures associated with maternal Western diet exposure. **(A)** Volcano plot showing differentially expressed genes (DEGs) in PBMCs from adult offspring of Western diet-fed dams (O-WD) relative to controls (O-C). Red dots indicate significantly upregulated genes, and gray dots represent non-significant genes (*p* < 0.05). **(B)** Partial Least Squares Discriminant Analysis (PLS-DA) scores plot showing the separation between O-C (green) and O-WD (yellow) animals based on global PBMC transcriptomic profiles. **(C)** Variable Importance in Projection (VIP) scores for the top 20 most discriminative transcripts identified by PLS-DA. **(D)** KEGG pathway enrichment analysis of the 298 DEGs identified between O-WD and O-C groups. Pathways are organized by category and subcategory. The *p*-value and adjusted *p*-value (Benjamini–Hochberg correction) are shown for each pathway, along with the number of DEGs mapped to each pathway and the corresponding gene symbols.

A total of 14,543 coding transcripts were analyzed. Differential gene expression analysis identified 298 differentially expressed genes (DEGs; *p* < 0.05) in O-WD animals compared to O-C animals. Of these, 155 genes were downregulated, and 143 were upregulated (Figure 1A; complete list in Supplementary Table 1). After correction for multiple comparisons, four DEGs remained statistically significant: *Ctrb1* (chymotrypsinogen B1, *p* < 0.001, padj = 0.001), *Krt15* (keratin 15, *p* < 0.001, padj = 0.007), *Scgb1a1* (secretoglobin family 1A member 1, *p* < 0.001, padj = 0.008), and *Clca1* (chloride channel accessory 1, *p* < 0.001, padj = 0.012).

The PLS-DA results, including the top 20 most discriminative variables, are shown in Figures 1B,C. This exploratory analysis showed a separation between both groups based on the overall transcriptomic profile. The model explained 39.8% of the variance (34% PC1 and 5.8 PC2) and showed a high goodness of fit for the response variable [*R*^2^Y(cum) = 0.927]. However, the predictive ability of the model was limited, as indicated by a low *Q*^2^(cum) value (0.06), suggesting potential overfitting. Permutation testing (*n* = 1,000) showed that while the *R*^2^Y value was statistically significant (p*R*^2^Y = 0.007), the *Q*^2^ value was not (p*Q*^2^ = 0.377), further supporting the lack of predictive reliability of the model. Therefore, the PLS-DA results should be interpreted in an exploratory context, reflecting global transcriptomic differences rather than a validated predictive classification model.

Notably, all VIP-selected markers showed higher mean expression levels in the O-WD group. Thirteen of the transcripts identified by PLS-DA also overlapped with DEGs, indicating partial concordance between the multivariate and univariate approaches. They included *Palmd* (palmdelphin, *p* < 0.001, padj = 0.582), *Traf5* (TNF receptor associated factor 5, *p* = 0.003, padj = 1.000), *Spib* (Spi-B transcription factor, *p* = 0.012,padj = 1.000), *Srpk3* (SRSF protein kinase 3, *p* = 0.035, padj = 1.000), *Tnfrsf13c* (TNF receptor superfamily member 13C, *p* = 0.03, padj = 1.000), *Spag1* (sperm associated antigen 1, *p* = 0.024, padj = 1.000), *Syvn1* (synoviolin 1, *p* = 0.003, padj = 1.000), *Fcer2* (Fc epsilon receptor II, *p* = 0.032, padj = 1.000), *Scd1* (stearoyl-CoA desaturase, *p* = 0.007, padj = 1.000), *Hes5* (hes family bHLH transcription factor 5, *p* = 0.025,padj = 1.000), *Peg3* (paternally expressed 3, *p* < 0.001, padj = 0.456), *Chst1* (carbohydrate sulfotransferase 1, *p* = 0.017, padj = 1.000), and *Swap70* (switching B-cell complex subunit SWAP70, *p* = 0.020, padj = 1.000). The remaining VIP-selected transcripts (*Gtse1* (G-2 and S-phase expressed 1, *p* = 0.162), *Chka* (choline kinase alpha, *p* = 0.068), *Fam3c* (family with sequence similarity 3, member C, *p* = 0.099), *Tmem206* (transmembrane protein 206, *p* = 0.131), *Pxdc1* (PX domain containing 1, *p* = 0.093), *Fam129c* (family with sequence similarity 129, member C, *p* = 0.053) and *Gypc* (glycophorin C, *p* = 0.054) were not identified as significantly different in the DESeq2 analysis.

Functional enrichment analysis of the 298 DEGs identified several enriched KEGG pathways (Figure 1D). Specifically, within the cellular processes category, necroptosis (rno04217, *p* = 0.001, padj = 0.303) was notably enriched. Similarly, in the environmental information processing category, the NF-kappa B signaling pathway (rno04064, *p* = 0.007, padj = 0.574), Cadherin signaling (rno04519, *p* = 0.017, padj = 0.574), ECM–receptor interaction (rno04512, *p* = 0.031, padj = 0.799), and Integrin signaling (rno04518, *p* = 0.048, padj = 0.799) were overrepresented based on nominal *p*-values. Although these pathways did not remain significant after multiple-testing correction, they were considered biologically relevant given the long-term nature of the programming effects under study. Collectively, these findings suggest coordinated alterations in immunometabolic and cell-signaling pathways associated with maternal WD exposure, even in the absence of overt phenotypic alterations in adulthood.

### 3.3 Transcriptomic impact of leptin supplementation and maternal diet improvement during lactation

**Figure 2:**
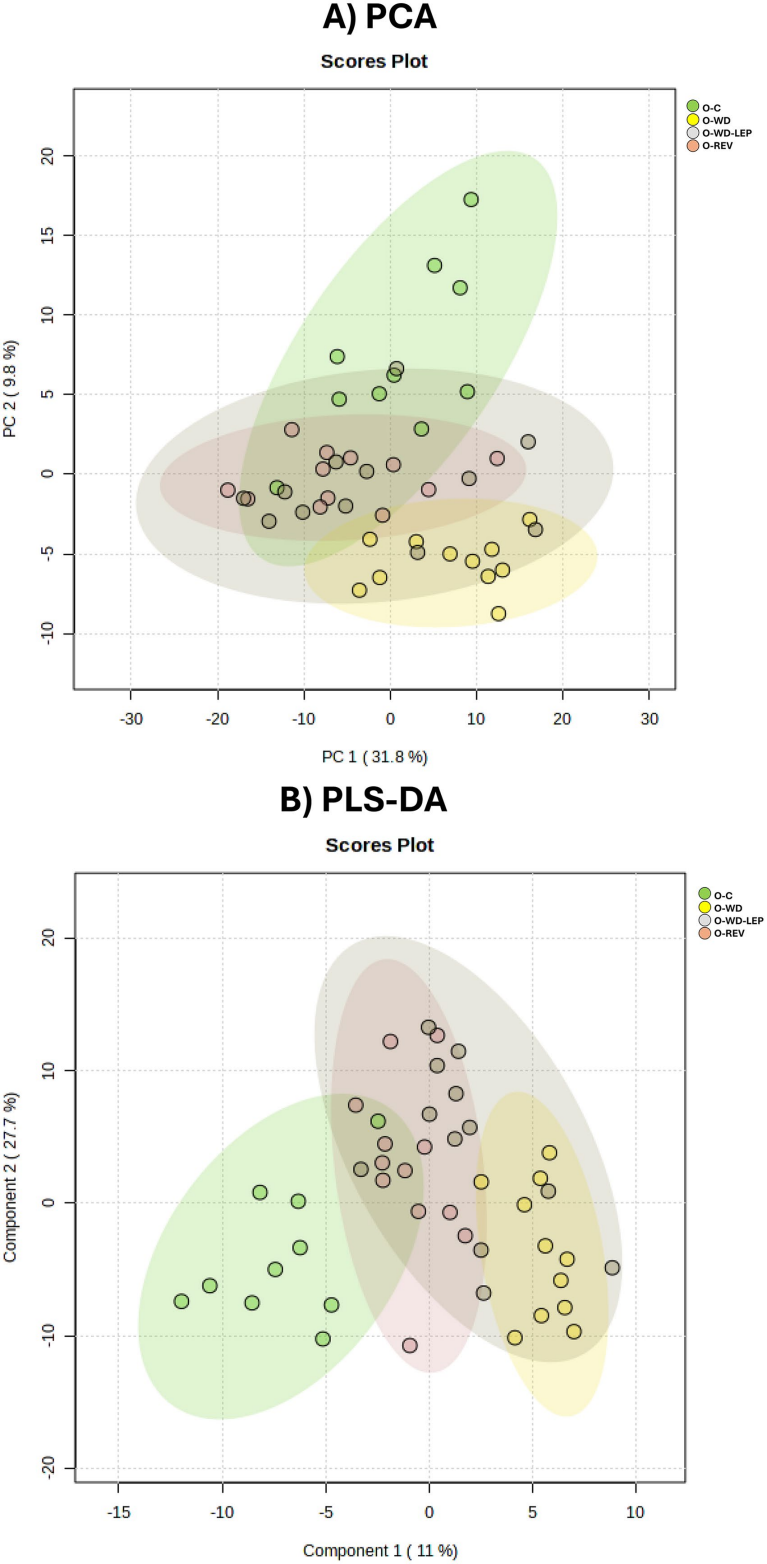
Principal Component Analysis (PCA) **(A)** and Partial Least Squares Discriminant Analysis (PLS-DA) **(B)** scores plots showing the distribution of individual samples across experimental groups based on PBMC transcriptomic profiles of the 298 differentially expressed genes identified between O-WD and O-C animals.

To assess whether leptin supplementation or maternal dietary improvement during lactation modulates the transcriptomic changes associated with maternal WD exposure, we examined the expression patterns of the 298 previously identified DEGs between O-C and O-WD groups, incorporating the O-WD-LEP and O-REV groups into the analysis (Figure 2). Principal Component Analysis (PCA) and PLS-DA showed that O-REV animals clustered between the O-C and O-WD groups, suggesting that maternal dietary improvement during lactation partially reversed WD-induced gene expression changes. A similar, albeit more variable, intermediate clustering was observed in O-WD-LEP animals, with two individuals clustered closely with the O-WD group. In the PCA, PC1 explained 31.8% and PC2 explained 9.8% of the total variance. In the PLS-DA, Component 1 accounted for 11% and Component 2 for 27.8% of the variance. The PLS-DA model showed a moderate fit to the data (*R*^2^Y(cum) = 0.352) and moderate predictive ability according to cross-validation (Q^2^(cum) = 0.275). Model robustness was further supported by permutation testing (*n* = 1,000), yielding significant values for both *R*^2^Y (p*R*^2^Y = 0.002) and Q^2^ (pQ^2^ = 0.001). Although the proportion of variance explained by the separating components is modest, this is consistent with the subtle and long-term transcriptomic effects expected in developmental programming studies, rather than the pronounced transcriptional changes typically associated with acute perturbations.

To determine whether necroptosis and NF-κB pathway genes contributed to the multivariate separation between groups, we examined their loadings on the PCA and PLS-DA components (Supplementary Table 3). Overall, these genes exhibited relatively modest loading values (|loading| < ~0.2) across both PCA and PLS-DA. This pattern suggests that their contribution to group separation is distributed across multiple genes rather than being driven by a small number of highly influential variables, consistent with coordinated but moderate alterations at the pathway level, rather than dominant effects of individual genes.

**Figure 3:**
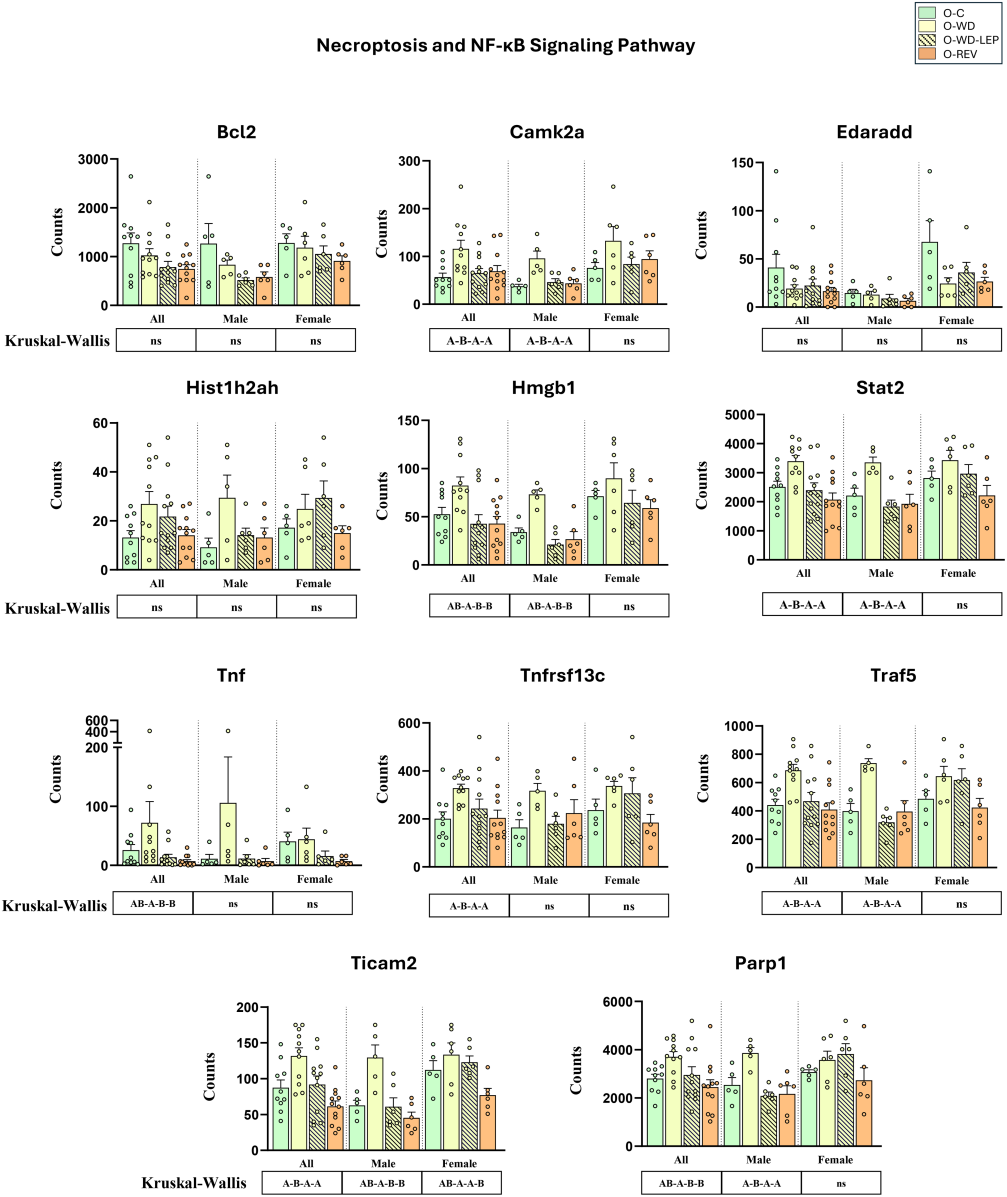
Expression levels of key genes within the Necroptosis and NF-κB signaling pathways in PBMCs from adult offspring across experimental groups. Bar graphs show read counts (mean ± SEM) for each gene, stratified by the total cohort (All), males, and females. Individual data points (circles) represent values from each animal (*n* = 5–6 in each group). Experimental groups are represented as follows: O-C (green bars), O-WD (yellow bars), O-WD-LEP (yellow hatched bars), and O-REV (orange bars). Statistical comparisons were performed using the Kruskal–Wallis test followed by post-hoc analysis. Different letters denote statistically significant differences between groups (*p* < 0.05); ns, not significant.

Based on the pathway-level enrichment results, we next focused on the necroptosis and NF-κB signaling pathways, which showed the most significant enrichment in O-WD animals. We compared expression levels of these genes across the O-C, O-WD, O-WD-LEP, and O-REV groups using the Kruskal-Wallis test followed by post-hoc analysis. As shown in Figure 3, *Camk2a*, *Stat2*, *Traf5*, *Tnfrsf13c*, and *Ticam2* were upregulated in O-WD animals compared to all other groups, with differences being particularly marked in males. This pattern suggests that both leptin supplementation and maternal dietary improvement during lactation were effective in normalizing the expression of these genes toward control levels. A second group of genes, including *Hmgb1*, *Tnf*, and *Parp1,* showed significantly higher expression in O-WD animals compared to the intervention groups, with no significant differences relative to controls. This pattern suggests that the interventions modulated the expression of these genes relative to O-WD animals, resulting in levels comparable to those observed in controls.

**Figure 4:**
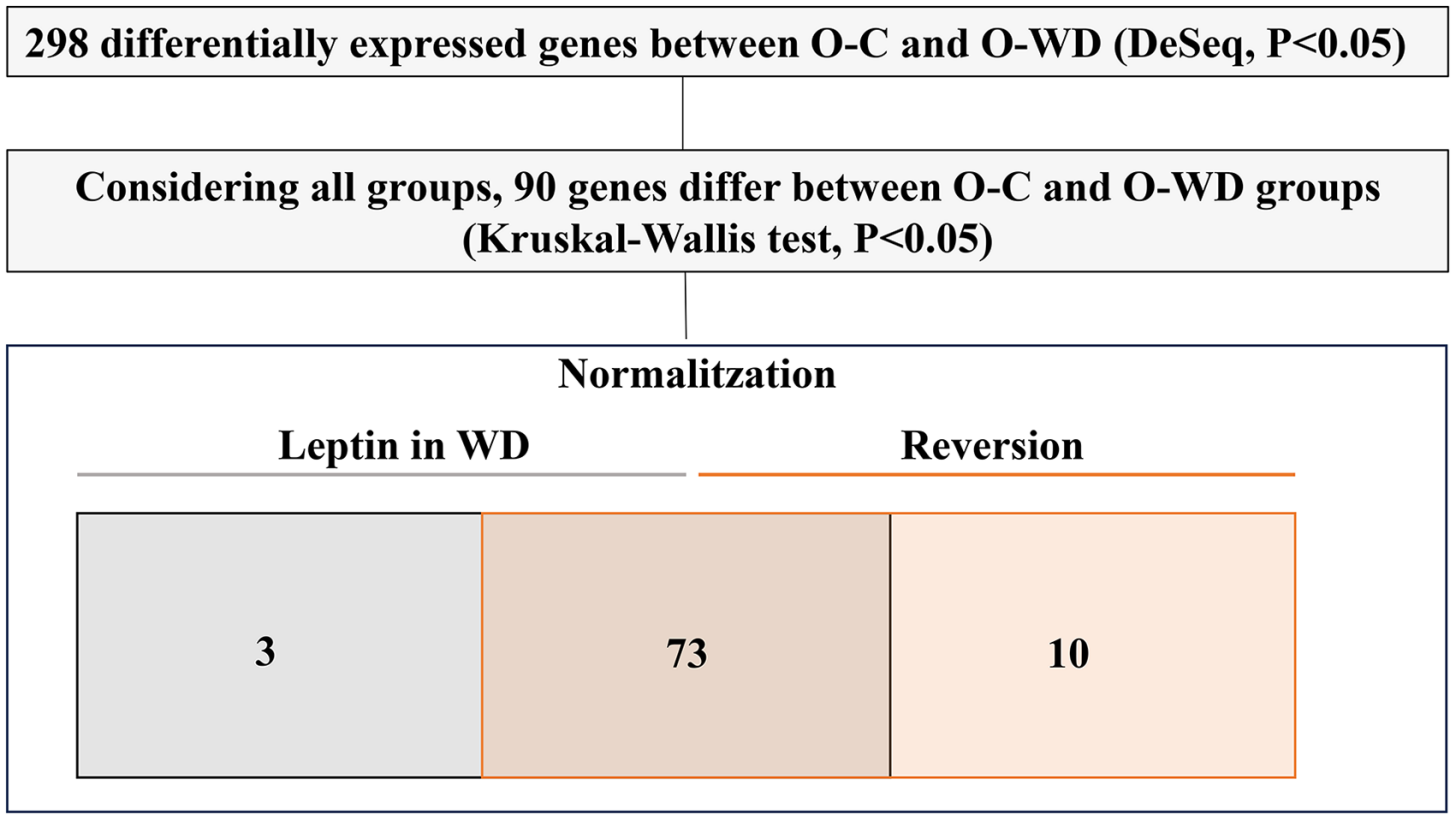
Summary of differentially expressed genes between O-C and O-WD and their normalization in response to postnatal interventions (leptin treatment or maternal diet normalization during lactation).

To globally assess transcriptomic recovery by interventions during lactation, a multi-group comparison was performed across the full set of DEGs (Supplementary Table 2; summary in Figure 4). Considering all experimental groups, 90 transcripts were significantly altered in O-WD animals compared to controls. Of these, leptin supplementation normalized the expression of 76, while maternal dietary improvement normalized 83, with 73 transcripts shared between both interventions.

Four genes -*Wnt10b, Dpt, Cfap54,* and *Ctrb1*- remained dysregulated in both intervention groups, following an A-B-B-B pattern (Supplementary Table 2). Specifically, *Wnt10b*, *Dpt*, and *Cfap54* remained downregulated, whereas *Ctrb1* remained upregulated in both O-WD-LEP and O-REV animals. In addition to these shared alterations, O-WD-LEP animals showed persistent dysregulation in the expression of *Plet1*, *Tle1*, *N4bp3*, *Scgb1a1*, *Bglap*, *Olr59*, *Fkbp9*, *Snta1*, *Ifi27* and *Upk1b* relative to controls, whereas O-REV animals showed persistent dysregulation in the expression of *Edn1*, *Prss8,* and *Enah* relative to controls. These findings identify a subset of transcripts that were unresponsive to either intervention, suggesting a persistent transcriptomic memory of early WD exposure.

**Figure 5:**
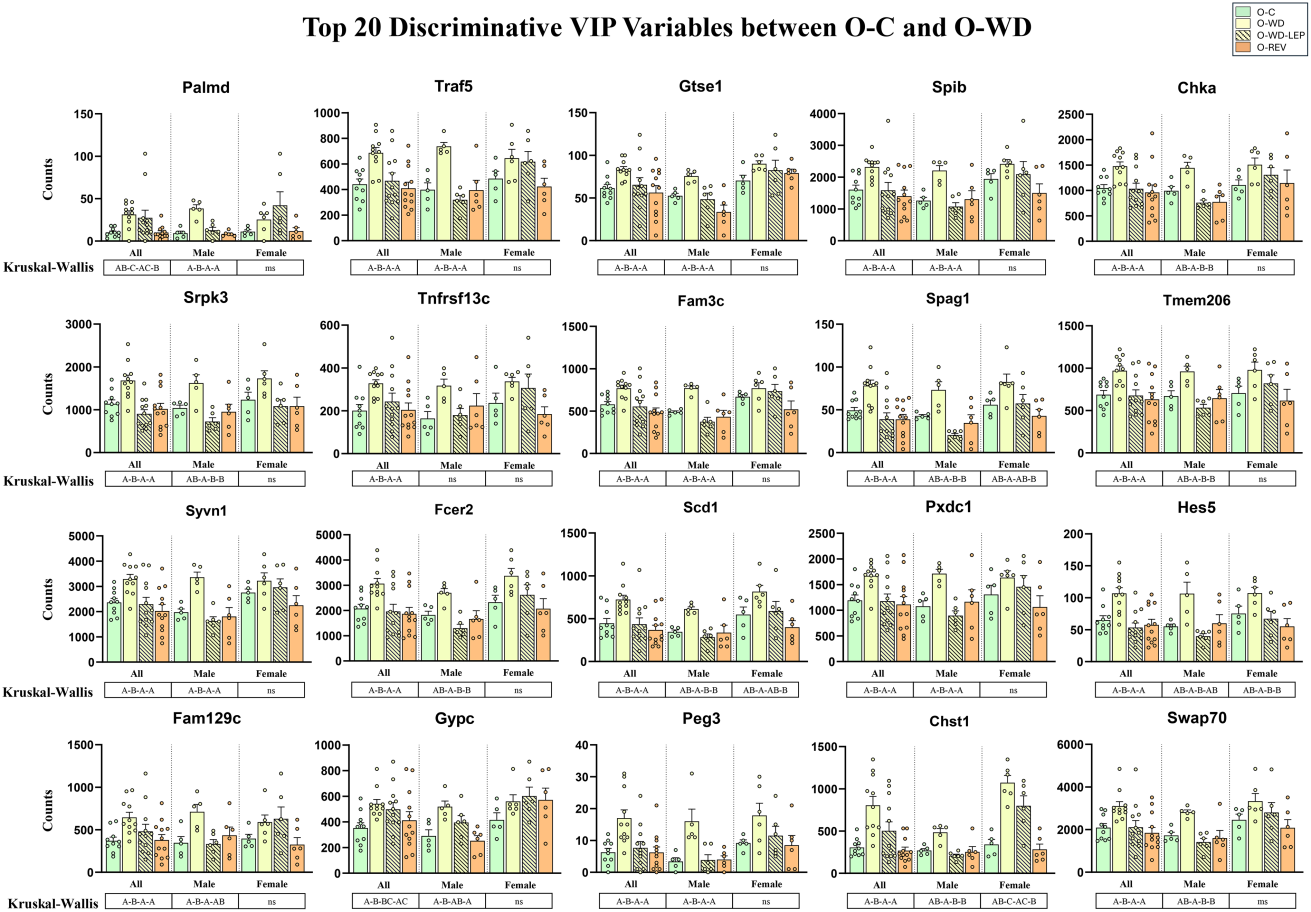
Expression levels of the top 20 most discriminative VIP transcripts, identified by comparing control (O-C) and Western diet (O-WD) groups in PBMCs, shown across all experimental groups (O-C, O-WD, O-WD-LEP, O-REV). Bar graphs represent normalized read counts (mean ± SEM) for each gene, stratified by the total cohort (All), males, and females. Individual data points (circles) indicate values from each animal (*n* = 5–6 in each group). Experimental groups are color-coded as follows: O-C (green bars), O-WD (yellow bars), O-WD-LEP (yellow hatched bars), and O-REV (orange bars). Statistical comparisons were performed using the Kruskal–Wallis test followed by post-hoc analysis. Different letters denote statistically significant differences between groups (*p* < 0.05); ns, not significant.

To identify potential biomarkers across all groups, we focused on the top 20 most discriminative VIP transcripts between O-C and O-WD animals. This analysis confirmed elevated expression of these transcripts in O-WD animals compared to controls (Figure 5). Notably, both interventions normalized the expression of most of these genes, including *Traf5, Gtse1, Spib, Chka, Sprk3, Tnfrsf13c, Fam3c, Spag1, Tmem206, Syvn1, Fcer2, Scd1, Pxdc1, Hes5, Fam129c, Peg3, Chst1,* and *Swap70,* which followed a characteristic A-B-A-A pattern, indicating increased expression in O-WD animals that returned to control-like levels in both intervention groups. Transcripts of *Palmd* and *Gypc* showed patterns primarily normalized by maternal dietary improvement (AB-C-AC-B, and A-B-BC-AC patterns, respectively).

**Figure 6:**
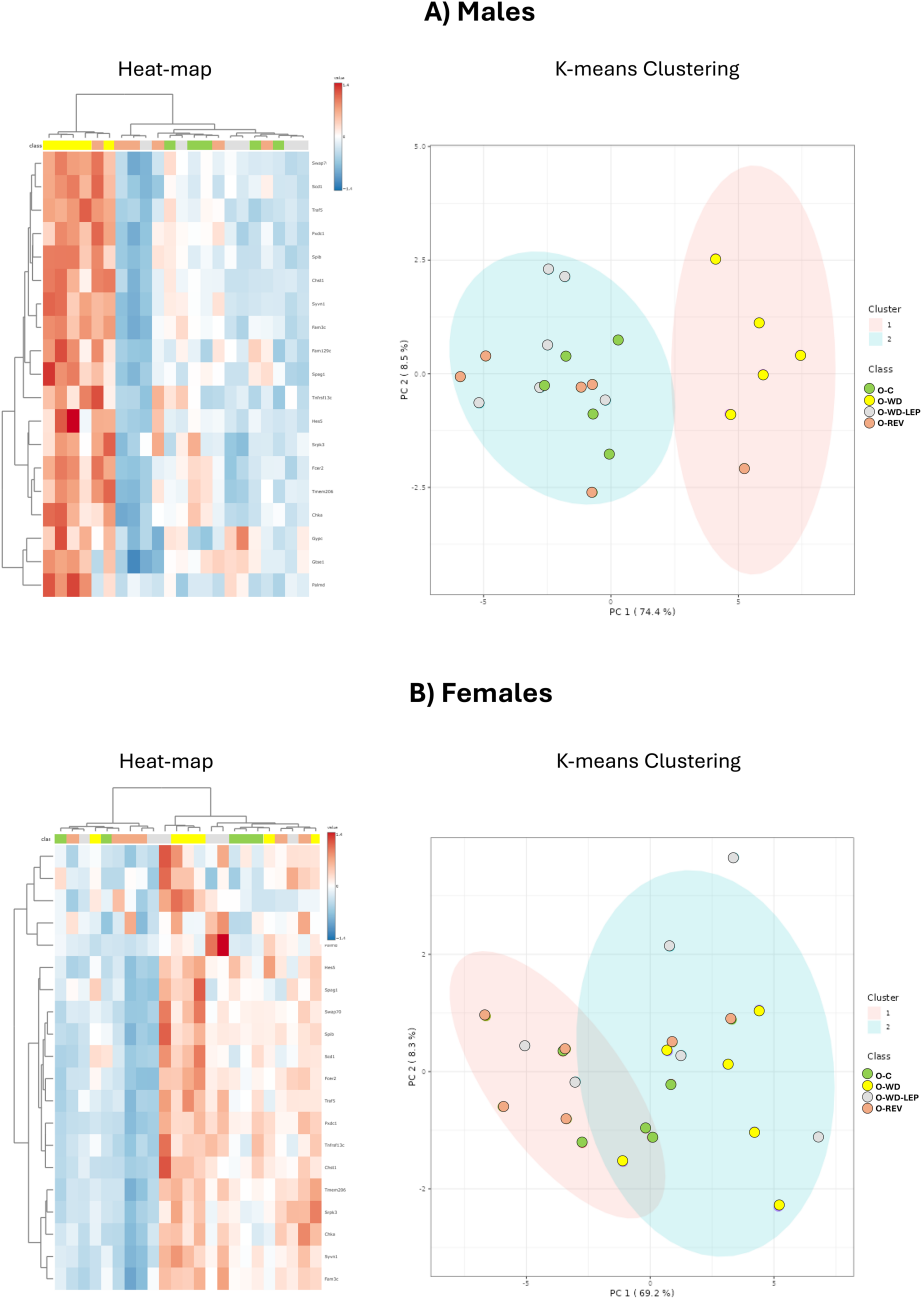
Heatmap and k-means clustering analysis of PBMC transcriptomic profiles in males **(A)** and females **(B)** across all experimental groups (O-C, O-WD, O-WD-LEP, O-REV), using the 20 most discriminative VIP transcripts identified by comparing O-C and O-WD groups.

When data analysis was stratified by sex, transcriptomic alterations were more pronounced in males. In fact, 16 of the 20 transcripts did not show significant group differences in females, indicating a predominantly male-biased response (Figure 5). Heatmap analysis and k-means clustering supported this sex-dependent pattern (Figure 6). In males, the 20-gene set separated O-WD animals from O-C, O-WD-LEP, and O-REV groups (Figure 6A). In contrast, the same gene set did not clearly discriminate O-WD females from their respective control and intervention groups (Figure 6B). These results indicate sex-specific differences in the transcriptomic response to maternal WD exposure, with stronger discriminatory patterns observed in males.

## 4 Discussion

There is increasing evidence that environmental exposures during critical developmental windows can exert long-lasting effects on health. Among these, maternal exposure to an obesogenic environment during gestation and lactation has emerged as an important determinant of offspring susceptibility to metabolic dysfunction later in life. In this context, the present study aimed to identify persistent transcriptomic signatures associated with early-life exposure to a maternal WD using PBMCs as an accessible source of systemic biomarkers. In addition, we evaluated whether two proposed postnatal interventions, maternal dietary normalization during lactation and pup leptin supplementation, both previously shown to improve metabolic health, can mitigate or reverse these transcriptomic alterations.

PBMCs continuously circulate throughout the body and are exposed to tissue-derived signals, hormones, and nutrients, enabling them to respond dynamically to changes in the internal environment (14, 24). As a result, they are considered “sentinel” cells that can reflect systemic physiological and pathological states and have been proposed as minimally invasive biomarkers of metabolic alterations. However, PBMC gene expression cannot be assumed to reproduce the transcriptional profile of a specific organ accurately. Therefore, our findings should be interpreted as reflecting systemic responses rather than organ-specific effects. In addition, since protein expression and functional outcomes were not assessed in the present study, the observed transcriptional changes cannot be directly extrapolated to downstream effects at the protein level.

Previous findings from the full cohort of animals demonstrated that maternal WD exposure was associated with adverse metabolic outcomes in the offspring, including increased body weight and metabolic alterations, which were largely reversed by maternal dietary normalization and also improved by neonatal leptin supplementation (7). However, in the subcohort analyzed in the present study, these phenotypic differences were attenuated, likely due to reduced sample size and experimental stratification.

Despite the absence of overt metabolic differences within the studied subcohort, persistent transcriptomic alterations were detected in PBMCs at 4 months of age in offspring born to WD-fed dams after exposure to a standard control diet from weaning to adulthood. This suggests that early nutritional exposure can induce long-lasting molecular programming that remains detectable even in the absence of overt metabolic alterations, underscoring the sensitivity of transcriptomic profiling as a tool to identify molecular signatures of developmental programming. The relatively small number of significant DEGs may reflect the subtle and long-lasting nature of the programming effects induced by maternal WD exposure (25), together with the limited statistical power associated with the modest sample size and inherent biological variability of transcriptomic studies.

Functional enrichment analyses revealed that genes altered by maternal WD exposure were clustered in pathways related to inflammation, immune regulation, and cellular stress responses, particularly necroptosis and NF-κB signaling. Necroptosis, a form of programmed cell death distinct from apoptosis and necrosis, has been associated with inflammatory conditions and related diseases (26). It promotes the release of intracellular danger signals and amplifies inflammatory responses (27, 28). The concurrent enrichment of NF-κB signaling further supports the involvement of inflammatory and cell survival pathways. Although NF-κB is best known for its antiapoptotic role, it also interacts with other cell fate-regulating processes, such as autophagy and necroptosis (29). These findings reinforce the proposed role of PBMCs as sentinels of systemic metabolic-inflammatory crosstalk (30) and are consistent with a sustained inflammatory transcriptomic signature due to maternal diet, which could reflect a greater predisposition to related alterations later on.

A key finding of this study is that the majority of transcriptomic changes induced by maternal WD exposure were normalized by interventions applied during lactation. Both maternal dietary improvement and neonatal leptin supplementation shifted the global gene expression profile toward that of control offspring, showing substantial overlap in the genes normalized. Maternal dietary normalization exerted a slightly greater overall effect, in line with previous phenotypic and metabolic findings (7, 8). These results highlight lactation as a critical postnatal window during which adverse prenatal programming can be at least partially reversed, reinforcing the notion of developmental plasticity beyond gestation.

The 20 highest-ranked contributors to the model distinguishing offspring of control and WD-fed dams provided further insight into the biological processes potentially associated with the long-term effects of maternal WD exposure. Notably, all these transcripts showed higher expression levels in O-WD animals. Among them, *Palmd* and *Gtse1* (the first and third VIP-ranked variables, respectively) suggest altered regulation of apoptosis, cell cycle progression, and B-cell homeostasis in the offspring of WD-fed dams. *Palmd* encodes a lipid-anchored protein involved in the regulation of cell shape (31) and has been implicated in cell fate decisions, particularly in the balance between cell survival and apoptosis (32). Under basal conditions, *PALMD* levels are low but detectable in the cytoplasm, a localization that appears to be required for cell survival (32). In response to DNA damage, p53 induces *PALMD* expression, leading to its cytoplasmic accumulation and subsequent nuclear translocation, where it promotes apoptotic cell death (32). The increased expression of *PALMD* observed in PBMCs of O-WD animals may therefore reflect altered regulation of pathways involved in cell survival and apoptosis. Likewise, *Gtse1*, a p53-inducible gene specifically expressed during the S and G2 phases of the cell cycle, was also overexpressed in offspring of WD-fed dams. In response to DNA damage, GTSE1 accumulates in the nucleus, where it downregulates p53 activity, thereby limiting apoptosis and promoting progression through the G2/M transition (33). Increased GTSE1 expression has been reported in several human malignancies, including hepatocellular carcinoma (34), supporting its role in the regulation of cell-cycle progression and cellular survival mechanisms.

Similarly, the altered expression of *SpiB*, *Traf5*, *Tnfrsf13c* (BAFFR), *Fcer2* (CD23), and *Swap70* points to long-lasting changes in pathways related to B-cell signaling and adaptive immune regulation. SpiB (Spi-B transcription factor) is a hematopoietic cell-specific transcription factor expressed in immune cell populations, including subsets of germinal center B cells and memory B cells, where it contributes to the maintenance of memory B cell populations (35). The lack of SpiB in germinal center and memory B cells results in reduced autophagy-related gene expression and activity, leading to mitochondrial ROS accumulation and increased susceptibility to apoptosis (35). In turn, *Traf5* (the second VIP-ranked variable) encodes a member of the tumor necrosis factor receptor-associated factor (TRAF) family, which mediates signal transduction from TNFR superfamily members and other immune receptors. TRAF proteins activate key downstream pathways, including nuclear factor κB (NF-κB) and mitogen-activated protein kinases (MAPKs), thereby regulating immune and inflammatory responses (36, 37). Experimental and clinical evidence suggest a protective role of TRAF5 in metabolic and inflammatory contexts. TRAF5 deficiency promotes inflammation and metabolic dysfunction in diet-induced obesity models, whereas in humans, its expression inversely correlates with obesity and is restored following bariatric surgery (38). In contrast to these observations, we detected increased *Traf5* expression in PBMCs from WD-exposed offspring. Notably, in animal models of diet-induced obesity, *Traf5* expression is markedly reduced in adipocytes and macrophages but increased in B cells (38). In B lymphocytes, TRAF5 negatively regulates Toll-like receptor signaling and downstream function (39), suggesting that its overexpression may reflect alterations in adaptive immune regulation. Consistent with this, other B cell–related genes, including *Tnfrsf13c* (encoding the BAFF receptor), *Fcer2* (CD23), and *Swap70*, were also differently expressed. These genes are involved in B-cell survival, differentiation, and immune function (40–43), collectively suggesting persistent modulation of adaptive immune pathways following early-life WD exposure.

Altered expression of *Chka*, *Fam3c*, *Syvn1*, and *Scd1* suggests that maternal WD exposure may also impact metabolic regulatory pathways. Choline kinase alpha (*Chka*), a key enzyme in phosphatidylcholine biosynthesis via the Kennedy pathway, has been linked to B cell survival and obesity-related metabolic alterations (44, 45). FAM3 metabolism regulating signaling molecule C (*Fam3c*), a hepatokine that suppresses hepatic gluconeogenesis, has been associated with improved insulin sensitivity and metabolic homeostasis (46, 47). In contrast, Synoviolin 1, the protein encoded by the *Syvn1* gene, a conserved multi-transmembrane ubiquitin ligase, has been implicated in lipid accumulation and inflammation in metabolic disease (48). Notably, its overexpression has been shown to significantly aggravate lipid accumulation and inflammation in non-alcoholic steatohepatitis disease (49). Finally, stearoyl-Coenzyme A desaturase 1 (SCD1), a rate-limiting enzyme in monounsaturated fatty acid synthesis, plays a central role in hepatic lipogenesis and adipogenesis, and its deficiency has been shown to protect against obesity, hepatic steatosis, and insulin resistance (50).

Overall, the transcriptomic alterations observed in the offspring of WD-fed dams suggest a coordinated modulation of immunometabolic pathways, encompassing immune and inflammatory signaling, cell fate regulation, and metabolic homeostasis, and are consistent with the presence of a persistent molecular signature of early-life nutritional exposure detectable in PBMCs. Consistent with these findings, our previous study using the full cohort (8) provided evidence of a pro-inflammatory state associated with maternal WD exposure. These effects, together with features of insulin resistance, were particularly evident or further exacerbated in offspring exposed to WD after weaning, highlighting the interaction between early-life programming and the postnatal dietary environment (7, 8). Notably, these alterations were reversed, at least in part, by maternal dietary normalization and neonatal leptin supplementation during lactation (7, 8). In line with this, most of the VIP variables differentiating the offspring of WD-fed dams were also normalized by one or both interventions, suggesting that these transcripts are responsive to postnatal nutritional modulation and may represent potential molecular indicators of early-life exposure effects.

In the context of DOHaD, altered maternal lipid metabolism, including changes in cholesterol and fatty acid composition, has been proposed as a relevant contributor to offspring metabolic programming (51, 52). Although maternal circulating lipids were not assessed in the present study, previous work in this animal model has shown that maternal WD exposure modifies the lipid composition of milk during lactation, including an increased arachidonic acid (AA) to eicosapentaenoic acid (EPA) plus docosahexaenoic acid (DHA) ratio in both maternal milk and offspring plasma (53). These changes were partially reversible following maternal dietary intervention during lactation (53). Together, these early-life lipid alterations may represent a potential upstream factor contributing to long-term immunometabolic programming in the offspring.

Despite the overall normalization induced by postnatal interventions, a limited subset of transcripts (*Wnt10b*, *Dpt*, *Cfap54*, and *Ctrb1*) remained persistently altered in both intervention groups. This suggests the persistence of a transcriptomic signature associated with early-life WD exposure despite postnatal intervention. In addition, sex-stratified analyses revealed that transcriptomic alterations were more pronounced in males. The 20 highest-ranked VIP contributors showed greater discriminatory capacity in males, distinguishing WD-exposed animals from controls and intervention groups, whereas discrimination between groups was less evident in females. This sexual dimorphism may reflect differential susceptibility to early nutritional insults and highlights the importance of considering sex as a biological variable in developmental programming studies. In this context, our findings further suggest that explicitly modeling sex in transcriptomic analyses may provide additional resolution for the interpretation of sex-specific effects.

The present study demonstrates that maternal consumption of an obesogenic diet during gestation and lactation is associated with long-lasting molecular changes in offspring PBMCs, which persist into adulthood despite dietary normalization after weaning and in the absence of overt metabolic alterations. At the transcriptomic level, these changes primarily involved coordinated alterations of immunometabolic pathways encompassing immune, inflammatory, apoptotic, and metabolic processes. Importantly, most transcriptomic alterations were reversed by interventions applied exclusively during lactation - maternal dietary improvement and neonatal leptin supplementation - which exhibited a substantial overlap in their effects on gene normalization, suggesting convergence on common molecular pathways involved in perinatal programming. This highlights lactation as a critical postnatal window of developmental plasticity to counteract the molecular consequences of adverse perinatal nutrition. These findings may have important translational relevance, as PBMC transcriptomic profiling offers a promising, minimally invasive approach to detect molecular signatures of metabolic risk. Future research should further evaluate these transcripts as potential biomarkers of metabolic imprinting and determine whether they are associated with long-term metabolic outcomes. Their apparent sensitivity compared with conventional metabolic parameters suggests potential utility as early indicators of subtle programming effects, although this requires validation in independent and longitudinal animal studies.

## Data Availability

The raw data supporting the conclusions of this article will be made available by the authors, without undue reservation.
