# Supplementary material for: Maternal Western diet programs persistent transcriptomic signatures in offspring PBMCs that are largely reversible by interventions during lactation
Source: Front Nutr. 2026 Jul 22;13:1892621. doi: 10.3389/fnut.2026.1892621 (PMC13440078; doi:10.3389/fnut.2026.1892621)
Supplement: Supplementary file 1 [file Table_1.DOCX]

**Supplementary Table 1.** Differentially expressed genes between O-C and O-WD animals identified by DESeq2. Columns indicate base mean, log₂ fold change (Log2FC), standard error (lfcSE), Wald test statistic (Stat), *p*-value, and adjusted *p*-value (Padj)

| **Gene**  **Symbol** | **Gene Name** | **Base**  **Mean** | **Log2FC** | **lfcSE** | **Stat** | **pValue** | **Padj** |
| --- | --- | --- | --- | --- | --- | --- | --- |
| **Bpifa1** | **BPI fold containing family A, member 1** | **6.40** | **-5.77** | **1.61** | **-3.58** | **<0.001** | **0.582** |
| **Krt15** | **keratin 15, type I** | **4.65** | **-5.57** | **1.14** | **-4.90** | **<0.001** | **0.007** |
| **Scgb1a1** | **secretoglobin, family 1A, member 1 (uteroglobin)** | **6.57** | **-5.53** | **1.15** | **-4.80** | **<0.001** | **0.008** |
| **Clca1** | **chloride channel accessory 1** | **8.63** | **-5.44** | **1.17** | **-4.65** | **<0.001** | **0.012** |
| **Pla2g4cl1** | **phospholipase A2, group IVC-like** | **4.82** | **-4.28** | **1.37** | **-3.12** | **0.002** | **1.000** |
| **Nog** | **noggin** | **2.50** | **-4.08** | **1.63** | **-2.51** | **0.012** | **1.000** |
| **Ccno** | **cyclin O** | **2.01** | **-4.05** | **1.21** | **-3.36** | **0.001** | **0.875** |
| **Krt13** | **keratin 13, type I** | **1.25** | **-3.94** | **1.87** | **-2.11** | **0.035** | **1.000** |
| **Vwa5a** | **von Willebrand factor A domain containing 5A** | **1.23** | **-3.87** | **1.41** | **-2.74** | **0.006** | **1.000** |
| **Krt4** | **keratin 4, type II** | **1.67** | **-3.77** | **1.70** | **-2.22** | **0.026** | **1.000** |
| **Plet1** | **placenta expressed transcript 1** | **1.62** | **-3.43** | **0.96** | **-3.56** | **<0.001** | **0.582** |
| **Upk1b** | **uroplakin 1B** | **0.76** | **-3.22** | **1.39** | **-2.32** | **0.021** | **1.000** |
| **Akr1c3** | **aldo-keto reductase family 1, member C3** | **2.11** | **-3.15** | **1.59** | **-1.98** | **0.048** | **1.000** |
| **Krt23** | **keratin 23, type I** | **1.08** | **-3.11** | **1.34** | **-2.31** | **0.021** | **1.000** |
| **Aqp5** | **Aquaporin-5** | **1.18** | **-2.94** | **1.30** | **-2.26** | **0.024** | **1.000** |
| **Slc39a4** | **solute carrier family 39 (zinc transporter), member 4** | **0.85** | **-2.66** | **1.17** | **-2.28** | **0.023** | **1.000** |
| **Atp13a4** | **ATPase type 13A4** | **1.34** | **-2.63** | **1.22** | **-2.15** | **0.031** | **1.000** |
| **Cyp2f4** | **cytochrome P450, family 2, subfamily f, polypeptide 4** | **1.77** | **-2.51** | **1.16** | **-2.17** | **0.030** | **1.000** |
| **Slc9a3** | **solute carrier family 9, subfamily A (NHE3, cation proton antiporter 3), member 3** | **1.06** | **-2.41** | **1.17** | **-2.05** | **0.040** | **1.000** |
| **Cd300ld** | **Cd300 molecule-like family member D** | **2.32** | **-2.4** | **1.06** | **-2.26** | **0.024** | **1.000** |
| **Chchd2** | **coiled-coil-helix-coiled-coil-helix domain containing 2** | **0.68** | **-2.37** | **1.16** | **-2.05** | **0.040** | **1.000** |
| **Ints1** | **integrator complex subunit 1** | **4.31** | **-2.34** | **0.89** | **-2.63** | **0.009** | **1.000** |
| **Zfp260** | **zinc finger protein 260** | **0.95** | **-2.29** | **1.00** | **-2.28** | **0.023** | **1.000** |
| **Tbcb** | **tubulin folding cofactor B** | **62.2** | **-2.28** | **0.80** | **-2.84** | **0.005** | **1.000** |
| **Nsun4** | **NOP2/Sun domain family, member 4** | **5.73** | **-2.21** | **0.71** | **-3.13** | **0.002** | **1.000** |
| **Ccdc27** | **coiled-coil domain containing 27** | **1.02** | **-2.15** | **1.07** | **-2.01** | **0.044** | **1.000** |
| **Olr288** | **olfactory receptor 288** | **1.11** | **-2.14** | **1.00** | **-2.13** | **0.033** | **1.000** |
| **Tac1** | **tachykinin, precursor 1** | **4.04** | **-2.12** | **0.66** | **-3.2** | **0.001** | **1.000** |
| **Gpha2** | **glycoprotein hormone alpha 2** | **2.65** | **-2.06** | **0.8** | **-2.59** | **0.010** | **1.000** |
| **Rhpn1** | **rhophilin, Rho GTPase binding protein 1** | **1.65** | **-2.04** | **0.97** | **-2.10** | **0.036** | **1.000** |
| **Ptprn2** | **protein tyrosine phosphatase, receptor type, N polypeptide 2** | **1.55** | **-2.03** | **0.86** | **-2.37** | **0.018** | **1.000** |
| **Wbp11** | **WW domain binding protein 11** | **22.4** | **-2.01** | **0.64** | **-3.13** | **0.002** | **1.000** |
| **Edn1** | **endothelin 1** | **17.9** | **-1.95** | **0.55** | **-3.52** | **<0.001** | **0.582** |
| **Myo1b** | **myosin Ib** | **5.42** | **-1.94** | **0.69** | **-2.83** | **0.005** | **1.000** |
| **Rgs8** | **regulator of G-protein signaling 8** | **2.97** | **-1.91** | **0.81** | **-2.35** | **0.019** | **1.000** |
| **Rln1** | **Prorelaxin 1 Relaxin B chain Relaxin A chain** | **4.57** | **-1.9** | **0.80** | **-2.38** | **0.017** | **1.000** |
| **Rmnd5b** | **required for meiotic nuclear division 5 homolog B** | **30.9** | **-1.87** | **0.46** | **-4.08** | **<0.001** | **0.133** |
| **Tnfsf15** | **tumor necrosis factor (ligand) superfamily, member 15** | **1.42** | **-1.81** | **0.82** | **-2.20** | **0.028** | **1.000** |
| **N4bp3** | **Nedd4 binding protein 3** | **6.92** | **-1.79** | **0.64** | **-2.81** | **0.005** | **1.000** |
| **Spag4** | **sperm associated antigen 4** | **3.65** | **-1.71** | **0.79** | **-2.16** | **0.031** | **1.000** |
| **Gpx8** | **glutathione peroxidase 8** | **1.96** | **-1.71** | **0.73** | **-2.34** | **0.019** | **1.000** |
| **Ndufa1** | **NADH dehydrogenase (ubiquinone) 1 alpha subcomplex, 1** | **27.4** | **-1.70** | **0.67** | **-2.54** | **0.011** | **1.000** |
| **Cacnb4** | **calcium channel, voltage-dependent, beta 4 subunit** | **16.7** | **-1.69** | **0.71** | **-2.39** | **0.017** | **1.000** |
| **Igf1** | **insulin-like growth factor 1** | **2.51** | **-1.67** | **0.76** | **-2.20** | **0.028** | **1.000** |
| **Ltk** | **leukocyte receptor tyrosine kinase** | **6.06** | **-1.66** | **0.63** | **-2.65** | **0.008** | **1.000** |
| **Kcnma1** | **potassium channel, calcium activated large conductance subfamily M alpha, member 1** | **2.31** | **-1.66** | **0.70** | **-2.36** | **0.018** | **1.000** |
| **Slc27a2** | **solute carrier family 27 (fatty acid transporter), member 2** | **174.4** | **-1.64** | **0.82** | **-2.00** | **0.045** | **1.000** |
| **Zfp111** | **zinc finger protein 111** | **3.22** | **-1.58** | **0.51** | **-3.11** | **0.002** | **1.000** |
| **Col2a1** | **collagen, type II, alpha 1** | **4.08** | **-1.57** | **0.75** | **-2.08** | **0.037** | **1.000** |
| **Ebna1bp2** | **EBNA1 binding protein 2** | **94.9** | **-1.56** | **0.75** | **-2.08** | **0.037** | **1.000** |
| **Ighg** | **Immunoglobulin heavy chain (gamma polypeptide)** | **7.22** | **-1.50** | **0.64** | **-2.35** | **0.019** | **1.000** |
| **Dpt** | **dermatopontin** | **167** | **-1.48** | **0.48** | **-3.07** | **0.002** | **1.000** |
| **Tspan1** | **tetraspanin 1** | **2.32** | **-1.47** | **0.69** | **-2.12** | **0.034** | **1.000** |
| **Adamts4** | **ADAM metallopeptidase with thrombospondin type 1 motif, 4** | **3.43** | **-1.45** | **0.63** | **-2.29** | **0.022** | **1.000** |
| **Dsp** | **desmoplakin** | **10.2** | **-1.44** | **0.61** | **-2.34** | **0.019** | **1.000** |
| **Ltbp2** | **latent transforming growth factor beta binding protein 2** | **5.36** | **-1.41** | **0.71** | **-2.00** | **0.046** | **1.000** |
| **Ankrd29** | **ankyrin repeat domain 29** | **2.23** | **-1.40** | **0.60** | **-2.34** | **0.019** | **1.000** |
| **Rasal2** | **RAS protein activator like 2** | **10.1** | **-1.36** | **0.48** | **-2.81** | **0.005** | **1.000** |
| **Pla2g2c** | **phospholipase A2, group IIC** | **3.47** | **-1.35** | **0.56** | **-2.42** | **0.016** | **1.000** |
| **Wnt10b** | **wingless-type MMTV integration site family, member 10B** | **4.14** | **-1.34** | **0.47** | **-2.89** | **0.004** | **1.000** |
| **Ramp2** | **receptor (G protein-coupled) activity modifying protein 2** | **5.31** | **-1.34** | **0.42** | **-3.21** | **0.001** | **1.000** |
| **Cfap54** | **cilia and flagella associated 54** | **3.84** | **-1.34** | **0.67** | **-2.00** | **0.045** | **1.000** |
| **Prss8** | **protease, serine, 8** | **5.18** | **-1.34** | **0.46** | **-2.92** | **0.003** | **1.000** |
| **Golga7b** | **golgin A7 family, member B** | **6.05** | **-1.30** | **0.62** | **-2.08** | **0.037** | **1.000** |
| **Nsun4** | **NOP2/Sun domain family, member 4** | **6.93** | **-1.27** | **0.55** | **-2.33** | **0.020** | **1.000** |
| **Duox2** | **dual oxidase 2** | **7.79** | **-1.22** | **0.46** | **-2.66** | **0.008** | **1.000** |
| **Cyp26b1** | **cytochrome P450, family 26, subfamily b, polypeptide 1** | **2.58** | **-1.21** | **0.58** | **-2.08** | **0.038** | **1.000** |
| **Lepr** | **leptin receptor** | **3.82** | **-1.20** | **0.55** | **-2.18** | **0.029** | **1.000** |
| **Enah** | **enabled homolog (Drosophila)** | **5.41** | **-1.20** | **0.44** | **-2.76** | **0.006** | **1.000** |
| **Gpat2** | **glycerol-3-phosphate acyltransferase 2, mitochondrial** | **11.4** | **-1.19** | **0.52** | **-2.28** | **0.023** | **1.000** |
| **Cysltr1** | **cysteinyl leukotriene receptor 1** | **10.2** | **-1.18** | **0.54** | **-2.21** | **0.027** | **1.000** |
| **Etnk2** | **ethanolamine kinase 2** | **7.07** | **-1.18** | **0.46** | **-2.54** | **0.011** | **1.000** |
| **Cd63** | **Cd63 molecule [** | **1158** | **-1.10** | **0.38** | **-2.93** | **0.003** | **1.000** |
| **Edaradd** | **EDAR-associated death domain** | **28.1** | **-1.10** | **0.48** | **-2.31** | **0.021** | **1.000** |
| **F12** | **coagulation factor XII (Hageman factor)** | **17.1** | **-1.09** | **0.54** | **-2.03** | **0.043** | **1.000** |
| **Mt2A** | **metallothionein 2A** | **33.4** | **-1.05** | **0.37** | **-2.85** | **0.004** | **1.000** |
| **Pcdhgb6** | **protocadherin gamma subfamily B, 6** | **10.9** | **-1.04** | **0.37** | **-2.79** | **0.005** | **1.000** |
| **Krt8** | **keratin 8, type II** | **8.45** | **-1.01** | **0.50** | **-2.01** | **0.044** | **1.000** |
| **Fbln1** | **fibulin 1** | **358** | **-0.99** | **0.41** | **-2.42** | **0.015** | **1.000** |
| **Myh1** | **myosin, heavy chain 1, skeletal muscle, adult** | **9.32** | **-0.96** | **0.42** | **-2.31** | **0.021** | **1.000** |
| **Ltc4s** | **leukotriene C4 synthase** | **11.5** | **-0.95** | **0.47** | **-2.02** | **0.044** | **1.000** |
| **Scin** | **scinderin** | **186** | **-0.93** | **0.47** | **-1.99** | **0.046** | **1.000** |
| **Coro2b** | **coronin, actin binding protein, 2B** | **83.9** | **-0.89** | **0.32** | **-2.74** | **0.006** | **1.000** |
| **Sulf1** | **sulfatase 1** | **25.6** | **-0.87** | **0.37** | **-2.37** | **0.018** | **1.000** |
| **Tmprss6** | **transmembrane protease, serine 6** | **8.08** | **-0.87** | **0.40** | **-2.17** | **0.030** | **1.000** |
| **Mmel1** | **membrane metallo-endopeptidase-like 1** | **25.6** | **-0.87** | **0.42** | **-2.06** | **0.039** | **1.000** |
| **Epb41l3** | **erythrocyte membrane protein band 4.1-like 3** | **5.71** | **-0.87** | **0.43** | **-2.01** | **0.045** | **1.000** |
| **Etv1** | **ets variant 1** | **12.8** | **-0.85** | **0.32** | **-2.62** | **0.009** | **1.000** |
| **Adap2** | **ArfGAP with dual PH domains 2** | **11.7** | **-0.85** | **0.34** | **-2.51** | **0.012** | **1.000** |
| **Cndp1** | **carnosine dipeptidase 1 (metallopeptidase M20 family)** | **10.1** | **-0.85** | **0.35** | **-2.40** | **0.017** | **1.000** |
| **Snip1** | **Smad nuclear interacting protein 1** | **25.5** | **-0.85** | **0.38** | **-2.23** | **0.026** | **1.000** |
| **Depdc7** | **DEP domain containing 7** | **5.23** | **-0.84** | **0.41** | **-2.03** | **0.042** | **1.000** |
| **Cacng4** | **calcium channel, voltage-dependent, gamma subunit 4** | **100** | **-0.83** | **0.41** | **-2.04** | **0.041** | **1.000** |
| **Arsi** | **arylsulfatase family, member I** | **42.5** | **-0.82** | **0.36** | **-2.26** | **0.024** | **1.000** |
| **Acacb** | **acetyl-CoA carboxylase beta** | **19.5** | **-0.82** | **0.32** | **-2.55** | **0.011** | **1.000** |
| **Npas2** | **neuronal PAS domain protein 2** | **16.2** | **-0.81** | **0.36** | **-2.25** | **0.024** | **1.000** |
| **Casq1** | **calsequestrin 1 (fast-twitch, skeletal muscle)** | **71.5** | **-0.80** | **0.36** | **-2.24** | **0.025** | **1.000** |
| **Plekhf1** | **pleckstrin homology domain containing, family F (with FYVE domain) member 1** | **50.5** | **-0.80** | **0.34** | **-2.36** | **0.018** | **1.000** |
| **Sash1** | **SAM and SH3 domain containing 1** | **8.38** | **-0.78** | **0.40** | **-1.97** | **0.049** | **1.000** |
| **Rassf6** | **Ras association (RalGDS/AF-6) domain family member 6** | **13.4** | **-0.76** | **0.27** | **-2.84** | **0.005** | **1.000** |
| **Gsdma** | **gasdermin A** | **12.5** | **-0.75** | **0.37** | **-2.04** | **0.041** | **1.000** |
| **Begain** | **brain-enriched guanylate kinase-associated** | **302** | **-0.69** | **0.29** | **-2.35** | **0.019** | **1.000** |
| **Gli1** | **GLI family zinc finger 1** | **15.9** | **-0.68** | **0.23** | **-3.01** | **0.003** | **1.000** |
| **Hdhd3** | **haloacid dehalogenase-like hydrolase domain containing 3** | **9.84** | **-0.66** | **0.31** | **-2.13** | **0.033** | **1.000** |
| **Rgs1** | **regulator of G-protein signaling 1** | **9779** | **-0.65** | **0.28** | **-2.36** | **0.018** | **1.000** |
| **Wfdc2** | **WAP four-disulfide core domain 2** | **26.4** | **-0.65** | **0.32** | **-2.04** | **0.041** | **1.000** |
| **Oplah** | **5-oxoprolinase (ATP-hydrolysing)** | **283.5** | **-0.65** | **0.31** | **-2.08** | **0.037** | **1.000** |
| **Prrt3** | **proline-rich transmembrane protein 3** | **15.9** | **-0.64** | **0.30** | **-2.11** | **0.035** | **1.000** |
| **Tgm1** | **transglutaminase 1** | **52.8** | **-0.64** | **0.30** | **-2.12** | **0.034** | **1.000** |
| **Mmrn2** | **multimerin 2** | **16.7** | **-0.62** | **0.27** | **-2.32** | **0.020** | **1.000** |
| **Apba1** | **amyloid beta (A4) precursor protein-binding, family A, member 1** | **61.4** | **-0.61** | **0.26** | **-2.30** | **0.021** | **1.000** |
| **Zfat** | **zinc finger and AT hook domain containing** | **432** | **-0.57** | **0.28** | **-2.04** | **0.041** | **1.000** |
| **Bcl2** | **B-cell CLL/lymphoma 2** | **1131** | **-0.57** | **0.27** | **-2.08** | **0.038** | **1.000** |
| **Vcan** | **versican** | **967** | **-0.56** | **0.28** | **-2.01** | **0.045** | **1.000** |
| **Thbs3** | **thrombospondin 3** | **29.6** | **-0.55** | **0.24** | **-2.25** | **0.024** | **1.000** |
| **Tp53i3** | **tumor protein p53 inducible protein 3** | **74.3** | **-0.54** | **0.17** | **-3.10** | **0.002** | **1.000** |
| **Socs1** | **suppressor of cytokine signaling 1** | **1268** | **-0.54** | **0.24** | **-2.27** | **0.023** | **1.000** |
| **Txnip** | **thioredoxin interacting protein** | **29614** | **-0.53** | **0.27** | **-1.97** | **0.049** | **1.000** |
| **Bhlha15** | **basic helix-loop-helix family, member a15** | **28.0** | **-0.53** | **0.25** | **-2.08** | **0.037** | **1.000** |
| **Nbn** | **nibrin** | **311** | **-0.52** | **0.20** | **-2.65** | **0.008** | **1.000** |
| **Dfna5** | **deafness, autosomal dominant 5 (human)** | **23.5** | **-0.52** | **0.23** | **-2.25** | **0.024** | **1.000** |
| **Als2cr12** | **amyotrophic lateral sclerosis 2 (juvenile) chromosome region, candidate 12** | **28.8** | **-0.52** | **0.23** | **-2.27** | **0.023** | **1.000** |
| **Prdm1** | **PR domain containing 1, with ZNF domain** | **548** | **-0.51** | **0.24** | **-2.09** | **0.036** | **1.000** |
| **Fam71b** | **family with sequence similarity 71, member B** | **38.0** | **-0.50** | **0.22** | **-2.29** | **0.022** | **1.000** |
| **She** | **Src homology 2 domain containing E** | **213** | **-0.48** | **0.22** | **-2.18** | **0.030** | **1.000** |
| **Arid5a** | **AT rich interactive domain 5A (Mrf1 like)** | **2979** | **-0.48** | **0.21** | **-2.31** | **0.021** | **1.000** |
| **Cep131** | **centrosomal protein 131** | **759** | **-0.47** | **0.20** | **-2.37** | **0.018** | **1.000** |
| **Hs3st1** | **heparan sulfate (glucosamine) 3-O-sulfotransferase 1** | **261** | **-0.47** | **0.19** | **-2.51** | **0.012** | **1.000** |
| **Slc36a3** | **solute carrier family 36, member 3** | **22.5** | **-0.47** | **0.21** | **-2.25** | **0.025** | **1.000** |
| **Irf6** | **interferon regulatory factor 6** | **399.1** | **-0.46** | **0.20** | **-2.37** | **0.018** | **1.000** |
| **Zfp605** | **zinc finger protein 605** | **17.7** | **-0.45** | **0.21** | **-2.12** | **0.034** | **1.000** |
| **Rpl39** | **ribosomal protein L39** | **36.9** | **-0.44** | **0.20** | **-2.17** | **0.030** | **1.000** |
| **Ctla4** | **Cytotoxic T-lymphocyte-associated protein 4, isoform CRA_b; Protein Ctla4** | **743** | **-0.43** | **0.15** | **-2.85** | **0.004** | **1.000** |
| **Slc26a10** | **solute carrier family 26, member 10** | **49.7** | **-0.42** | **0.18** | **-2.42** | **0.016** | **1.000** |
| **Trib2** | **tribbles pseudokinase 2** | **6020** | **-0.42** | **0.15** | **-2.70** | **0.007** | **1.000** |
| **Fhl2** | **four and a half LIM domains 2** | **237** | **-0.42** | **0.17** | **-2.44** | **0.015** | **1.000** |
| **Art2b** | **ADP-ribosyltransferase 2b** | **2565** | **-0.39** | **0.20** | **-1.97** | **0.049** | **1.000** |
| **Pc** | **pyruvate carboxylase** | **110** | **-0.39** | **0.14** | **-2.84** | **0.004** | **1.000** |
| **Adrbk2** | **adrenergic receptor kinase, beta 2** | **109** | **-0.38** | **0.18** | **-2.13** | **0.033** | **1.000** |
| **Plcb4** | **phospholipase C, beta 4** | **198** | **-0.38** | **0.18** | **-2.05** | **0.040** | **1.000** |
| **P2ry2** | **purinergic receptor P2Y, G-protein coupled, 2** | **142** | **-0.38** | **0.16** | **-2.43** | **0.015** | **1.000** |
| **Cycs** | **cytochrome c, somatic** | **106** | **-0.34** | **0.14** | **-2.36** | **0.018** | **1.000** |
| **Eya2** | **EYA transcriptional coactivator and phosphatase 2** | **173** | **-0.34** | **0.16** | **-2.08** | **0.038** | **1.000** |
| **Ifitm1** | **interferon induced transmembrane protein 1** | **2866** | **-0.31** | **0.13** | **-2.35** | **0.019** | **1.000** |
| **Slc39a13** | **solute carrier family 39 (zinc transporter), member 13** | **210** | **-0.30** | **0.14** | **-2.09** | **0.037** | **1.000** |
| **Socs2** | **suppressor of cytokine signaling 2** | **525** | **-0.29** | **0.14** | **-2.09** | **0.036** | **1.000** |
| **Mtmr10** | **myotubularin related protein 10** | **280** | **-0.29** | **0.14** | **-2.09** | **0.036** | **1.000** |
| **Rab7b** | **Rab7b, member RAS oncogene family** | **148** | **-0.27** | **0.13** | **-2.08** | **0.037** | **1.000** |
| **Vwa9** | **von Willebrand factor A domain containing 9** | **545** | **-0.24** | **0.09** | **-2.71** | **0.007** | **1.000** |
| **Eef2k** | **eukaryotic elongation factor-2 kinase** | **1085** | **-0.20** | **0.10** | **-2.00** | **0.046** | **1.000** |
| **Slc30a1** | **solute carrier family 30 (zinc transporter), member 1** | **391** | **-0.19** | **0.09** | **-2.19** | **0.028** | **1.000** |
| **Spata6** | **spermatogenesis associated 6** | **447** | **-0.17** | **0.06** | **-2.76** | **0.006** | **1.000** |
| **Tle3** | **transducin-like enhancer of split 3** | **4019** | **-0.14** | **0.06** | **-2.16** | **0.031** | **1.000** |
| **Ezr** | **ezrin** | **15041** | **-0.12** | **0.06** | **-2.03** | **0.042** | **1.000** |
| **Hnrnpf** | **heterogeneous nuclear ribonucleoprotein F** | **11102** | **-0.09** | **0.04** | **-2.04** | **0.041** | **1.000** |
| **Tial1** | **Tia1 cytotoxic granule-associated RNA binding protein-like 1** | **1827** | **0.11** | **0.05** | **2.23** | **0.025** | **1.000** |
| **Slc11a2** | **solute carrier family 11 (proton-coupled divalent metal ion transporter), member 2** | **530** | **0.12** | **0.06** | **2.09** | **0.036** | **1.000** |
| **Arhgap17** | **Rho GTPase activating protein 17** | **4231** | **0.12** | **0.06** | **2.12** | **0.034** | **1.000** |
| **Gba2** | **glucosidase beta 2** | **1695** | **0.13** | **0.06** | **2.27** | **0.023** | **1.000** |
| **Parp1** | **poly (ADP-ribose) polymerase 1** | **3188** | **0.17** | **0.07** | **2.53** | **0.011** | **1.000** |
| **Tmc8** | **transmembrane channel-like 8** | **4269** | **0.17** | **0.07** | **2.33** | **0.020** | **1.000** |
| **Mcm3** | **minichromosome maintenance complex component 3** | **2188** | **0.18** | **0.08** | **2.35** | **0.019** | **1.000** |
| **Papd7** | **PAP associated domain containing 7** | **888** | **0.19** | **0.09** | **1.99** | **0.047** | **1.000** |
| **Mrpl51** | **mitochondrial ribosomal protein L51** | **200** | **0.19** | **0.09** | **2.01** | **0.045** | **1.000** |
| **Dnaaf5** | **dynein, axonemal, assembly factor 5** | **222** | **0.20** | **0.08** | **2.39** | **0.017** | **1.000** |
| **Ttc37** | **tetratricopeptide repeat domain 37** | **1209** | **0.20** | **0.10** | **1.98** | **0.048** | **1.000** |
| **Sqle** | **squalene epoxidase** | **271** | **0.21** | **0.09** | **2.39** | **0.017** | **1.000** |
| **Med18** | **mediator complex subunit 18** | **478** | **0.21** | **0.11** | **2.01** | **0.044** | **1.000** |
| **Stat2** | **signal transducer and activator of transcription 2** | **2897** | **0.21** | **0.09** | **2.28** | **0.022** | **1.000** |
| **Tor4a** | **torsin family 4, member A** | **853** | **0.24** | **0.09** | **2.68** | **0.007** | **1.000** |
| **Srsf9** | **serine/arginine-rich splicing factor 9** | **644** | **0.24** | **0.10** | **2.37** | **0.018** | **1.000** |
| **Dimt1** | **DIM1 dimethyladenosine transferase 1 homolog** | **301** | **0.24** | **0.11** | **2.16** | **0.030** | **1.000** |
| **Syvn1** | **synovial apoptosis inhibitor 1, synoviolin** | **2777** | **0.24** | **0.08** | **2.92** | **0.004** | **1.000** |
| **Zfp467** | **zinc finger protein 467** | **525** | **0.25** | **0.11** | **2.25** | **0.025** | **1.000** |
| **Irak3** | **interleukin-1 receptor-associated kinase 3** | **1488** | **0.26** | **0.13** | **2.00** | **0.045** | **1.000** |
| **Smad1** | **SMAD family member 1** | **203** | **0.26** | **0.12** | **2.15** | **0.031** | **1.000** |
| **Slfn2** | **schlafen 2** | **9947** | **0.26** | **0.12** | **2.14** | **0.032** | **1.000** |
| **Mtfmt** | **mitochondrial methionyl-tRNA formyltransferase** | **111** | **0.26** | **0.11** | **2.40** | **0.016** | **1.000** |
| **Slc12a4** | **solute carrier family 12 (potassium/chloride transporter), member 4** | **474** | **0.26** | **0.10** | **2.74** | **0.006** | **1.000** |
| **Acsl3** | **acyl-CoA synthetase long-chain family member 3** | **235** | **0.28** | **0.14** | **1.99** | **0.047** | **1.000** |
| **Blk** | **BLK proto-oncogene, Src family tyrosine kinase** | **646** | **0.29** | **0.14** | **2.00** | **0.045** | **1.000** |
| **Hhex** | **hematopoietically expressed homeobox** | **528** | **0.30** | **0.15** | **1.99** | **0.047** | **1.000** |
| **Lamc1** | **laminin, gamma 1** | **257** | **0.30** | **0.15** | **2.01** | **0.044** | **1.000** |
| **Rslcan18** | **regulator of sex-limitation candidate 18** | **136** | **0.31** | **0.16** | **1.98** | **0.048** | **1.000** |
| **Lsr** | **lipolysis stimulated lipoprotein receptor** | **121** | **0.33** | **0.14** | **2.33** | **0.020** | **1.000** |
| **Klhl14** | **kelch-like family member 14** | **209** | **0.33** | **0.16** | **2.11** | **0.035** | **1.000** |
| **Cdkn1c** | **CDKI protein long isoform; Cyclin-dependent kinase inhibitor 1C (P57)** | **37.43** | **0.34** | **0.16** | **2.08** | **0.038** | **1.000** |
| **Fcrl1** | **Fc receptor-like 1** | **892.31** | **0.34** | **0.17** | **2.07** | **0.039** | **1.000** |
| **Pagr1** | **Paxip1-associated glutamate-rich protein 1** | **390.8** | **0.34** | **0.14** | **2.43** | **0.015** | **1.000** |
| **Spon1** | **spondin 1, extracellular matrix protein** | **32.60** | **0.35** | **0.18** | **1.96** | **0.050** | **1.000** |
| **Fcer2** | **Fc fragment of IgE, low affinity II, receptor for (CD23)** | **2565.53** | **0.35** | **0.16** | **2.14** | **0.033** | **1.000** |
| **Spib** | **Spi-B transcription factor (Spi-1/PU.1 related)** | **1949.81** | **0.35** | **0.14** | **2.50** | **0.012** | **1.000** |
| **Ms4a1** | **membrane-spanning 4-domains, subfamily A, member 1** | **1531.55** | **0.36** | **0.16** | **2.20** | **0.028** | **1.000** |
| **Rps6ka5** | **ribosomal protein S6 kinase, polypeptide 5** | **722.26** | **0.36** | **0.16** | **2.28** | **0.022** | **1.000** |
| **Swap70** | **SWAP switching B-cell complex 70** | **2584.25** | **0.37** | **0.16** | **2.33** | **0.020** | **1.000** |
| **Ticam2** | **toll-like receptor adaptor molecule 2** | **105.07** | **0.37** | **0.14** | **2.74** | **0.006** | **1.000** |
| **Myo1e** | **myosin IE** | **398.50** | **0.38** | **0.18** | **2.11** | **0.035** | **1.000** |
| **Sox12** | **SRY (sex determining region Y)-box 12** | **107.62** | **0.39** | **0.15** | **2.58** | **0.010** | **1.000** |
| **Lipt2** | **lipoyl(octanoyl) transferase 2 (putative)** | **47.38** | **0.39** | **0.19** | **2.12** | **0.034** | **1.000** |
| **Faim3** | **Fas apoptotic inhibitory molecule 3** | **5334.52** | **0.39** | **0.19** | **2.02** | **0.043** | **1.000** |
| **Fbxo39** | **F-box protein 39** | **43.79** | **0.39** | **0.19** | **2.08** | **0.037** | **1.000** |
| **Pask** | **PAS domain containing serine/threonine kinase** | **49.86** | **0.40** | **0.18** | **2.21** | **0.027** | **1.000** |
| **Kif22** | **kinesin family member 22** | **81.30** | **0.40** | **0.19** | **2.12** | **0.034** | **1.000** |
| **Chst1** | **carbohydrate (keratan sulfate Gal-6) sulfotransferase 1** | **384.04** | **0.41** | **0.17** | **2.40** | **0.017** | **1.000** |
| **Traf5** | **TNF receptor-associated factor 5** | **555.13** | **0.42** | **0.14** | **3.00** | **0.003** | **1.000** |
| **Id3** | **inhibitor of DNA binding 3** | **286.26** | **0.42** | **0.17** | **2.54** | **0.011** | **1.000** |
| **Spag1** | **sperm associated antigen 1** | **63.93** | **0.43** | **0.19** | **2.25** | **0.024** | **1.000** |
| **Srpk3** | **serine/arginine-rich protein specific kinase 3** | **382.69** | **0.43** | **0.20** | **2.11** | **0.035** | **1.000** |
| **Tonsl** | **tonsoku-like, DNA repair protein** | **58.28** | **0.44** | **0.21** | **2.05** | **0.041** | **1.000** |
| **Slfnl1** | **schlafen-like 1** | **28.81** | **0.45** | **0.17** | **2.58** | **0.010** | **1.000** |
| **Hmgb1** | **high mobility group box 1** | **64.86** | **0.45** | **0.20** | **2.27** | **0.023** | **1.000** |
| **Olfml3** | **olfactomedin-like 3** | **26.87** | **0.45** | **0.22** | **2.03** | **0.043** | **1.000** |
| **Ache** | **acetylcholinesterase** | **273.88** | **0.46** | **0.20** | **2.23** | **0.026** | **1.000** |
| **Caskin2** | **cask-interacting protein 2** | **40.7** | **0.48** | **0.22** | **2.15** | **0.032** | **1.000** |
| **Pcbd1** | **pterin-4 alpha-carbinolamine dehydratase/dimerization cofactor of hepatocyte nuclear factor 1 alpha** | **16.84** | **0.49** | **0.21** | **2.30** | **0.022** | **1.000** |
| **Lamc3** | **laminin gamma 3** | **26.25** | **0.49** | **0.24** | **2.03** | **0.042** | **1.000** |
| **A4gnt** | **alpha-1,4-N-acetylglucosaminyltransferase** | **18.85** | **0.49** | **0.25** | **1.99** | **0.047** | **1.000** |
| **Ctf1** | **cardiotrophin 1** | **24.11** | **0.50** | **0.21** | **2.34** | **0.019** | **1.000** |
| **Homez** | **homeobox and leucine zipper encoding** | **26.34** | **0.50** | **0.23** | **2.16** | **0.031** | **1.000** |
| **Ewsr1** | **EWS RNA-binding protein 1** | **202.71** | **0.51** | **0.25** | **2.04** | **0.041** | **1.000** |
| **Scd1** | **stearoyl-Coenzyme A desaturase 1** | **582.64** | **0.51** | **0.19** | **2.69** | **0.007** | **1.000** |
| **Cr2** | **complement component (3d/Epstein Barr virus) receptor 2** | **2021.12** | **0.52** | **0.25** | **2.07** | **0.038** | **1.000** |
| **Chst3** | **carbohydrate (chondroitin 6) sulfotransferase 3** | **394.25** | **0.52** | **0.22** | **2.32** | **0.020** | **1.000** |
| **Prrt2** | **proline-rich transmembrane protein 2** | **61.83** | **0.53** | **0.21** | **2.46** | **0.014** | **1.000** |
| **Hes5** | **hes family bHLH transcription factor 5** | **87.77** | **0.53** | **0.24** | **2.24** | **0.025** | **1.000** |
| **Gtpbp6** | **GTP binding protein 6 (putative)** | **227.73** | **0.53** | **0.23** | **2.36** | **0.018** | **1.000** |
| **Tnfrsf13c** | **tumor necrosis factor receptor superfamily, member 13c** | **259.59** | **0.54** | **0.18** | **3.00** | **0.003** | **1.000** |
| **Rpl7a** | **ribosomal protein L7a** | **1569.05** | **0.54** | **0.27** | **1.98** | **0.048** | **1.000** |
| **Kif23** | **kinesin family member 23** | **127.35** | **0.54** | **0.21** | **2.65** | **0.008** | **1.000** |
| **Slc52a3** | **solute carrier family 52, riboflavin transporter, member 3** | **28.78** | **0.57** | **0.26** | **2.22** | **0.026** | **1.000** |
| **Lzts1** | **leucine zipper, putative tumor suppressor 1** | **56.62** | **0.58** | **0.23** | **2.48** | **0.013** | **1.000** |
| **Odf3b** | **outer dense fiber of sperm tails 3B** | **33.23** | **0.59** | **0.29** | **2.02** | **0.043** | **1.000** |
| **Zdhhc2** | **zinc finger, DHHC-type containing 2** | **71.96** | **0.59** | **0.21** | **2.76** | **0.006** | **1.000** |
| **Acat2** | **acetyl-CoA acetyltransferase 2** | **129.51** | **0.59** | **0.21** | **2.83** | **0.005** | **1.000** |
| **Nbl1** | **neuroblastoma 1, DAN family BMP antagonist** | **22.11** | **0.61** | **0.29** | **2.07** | **0.038** | **1.000** |
| **Dok4** | **docking protein 4** | **126.97** | **0.63** | **0.32** | **2.00** | **0.046** | **1.000** |
| **Parp16** | **poly (ADP-ribose) polymerase family, member 16** | **25.12** | **0.64** | **0.22** | **2.99** | **0.003** | **1.000** |
| **Asb2** | **Ankyrin repeat and SOCS box protein 2** | **23.31** | **0.66** | **0.32** | **2.07** | **0.038** | **1.000** |
| **Lin7b** | **lin-7 homolog B** | **19.24** | **0.66** | **0.25** | **2.62** | **0.009** | **1.000** |
| **Creb3l1** | **cAMP responsive element binding protein 3-like 1** | **13.41** | **0.67** | **0.27** | **2.42** | **0.015** | **1.000** |
| **Epdr1** | **ependymin related 1** | **59.7** | **0.67** | **0.32** | **2.07** | **0.039** | **1.000** |
| **P4ha3** | **prolyl 4-hydroxylase, alpha polypeptide III** | **12.4** | **0.68** | **0.31** | **2.18** | **0.030** | **1.000** |
| **Slc13a4** | **solute carrier family 13 (sodium/sulfate symporter), member 4** | **76.33** | **0.69** | **0.34** | **2.04** | **0.041** | **1.000** |
| **Prr36** | **proline rich 36** | **30.92** | **0.69** | **0.26** | **2.61** | **0.009** | **1.000** |
| **Cidec** | **cell death-inducing DFFA-like effector c** | **24.4** | **0.69** | **0.32** | **2.17** | **0.030** | **1.000** |
| **Pgf** | **placental growth factor** | **17.31** | **0.7** | **0.31** | **2.23** | **0.026** | **1.000** |
| **Cd276** | **Cd276 molecule** | **14.58** | **0.71** | **0.33** | **2.19** | **0.029** | **1.000** |
| **Ak9** | **adenylate kinase 9** | **24.89** | **0.73** | **0.35** | **2.12** | **0.034** | **1.000** |
| **Xk** | **X-linked Kx blood group** | **425.06** | **0.75** | **0.27** | **2.76** | **0.006** | **1.000** |
| **Camk2a** | **calcium/calmodulin-dependent protein kinase II alpha** | **82.74** | **0.8** | **0.26** | **3.14** | **0.002** | **1.000** |
| **Hist1h2ah** | **histone cluster 1, H2ah** | **19.24** | **0.81** | **0.41** | **1.97** | **0.048** | **1.000** |
| **Sfrp5** | **secreted frizzled-related protein 5** | **12.64** | **0.82** | **0.38** | **2.17** | **0.030** | **1.000** |
| **Khdrbs3** | **KH domain containing, RNA binding, signal transduction associated 3** | **74.95** | **0.83** | **0.31** | **2.66** | **0.008** | **1.000** |
| **Sbk1** | **SH3 domain binding kinase 1** | **25.69** | **0.84** | **0.35** | **2.40** | **0.016** | **1.000** |
| **Triap1** | **TP53 regulated inhibitor of apoptosis 1** | **24.38** | **0.85** | **0.42** | **2.02** | **0.043** | **1.000** |
| **Hdac6** | **histone deacetylase 6** | **31.65** | **0.86** | **0.44** | **1.97** | **0.049** | **1.000** |
| **Kctd19** | **potassium channel tetramerization domain containing 19** | **5.64** | **0.86** | **0.38** | **2.26** | **0.024** | **1.000** |
| **Lama4** | **laminin, alpha 4** | **10.59** | **0.88** | **0.44** | **2.00** | **0.046** | **1.000** |
| **Snta1** | **syntrophin, alpha 1** | **31.54** | **0.88** | **0.31** | **2.80** | **0.005** | **1.000** |
| **Vps37c** | **vacuolar protein sorting 37 homolog C** | **55.62** | **0.99** | **0.39** | **2.54** | **0.011** | **1.000** |
| **Ifi27** | **interferon, alpha-inducible protein 27** | **4960.48** | **1.01** | **0.39** | **2.59** | **0.010** | **1.000** |
| **Pcdhb19** | **protocadherin beta 19** | **5.25** | **1.02** | **0.52** | **1.96** | **0.049** | **1.000** |
| **Olr59** | **olfactory receptor 59** | **7.22** | **1.06** | **0.46** | **2.29** | **0.022** | **1.000** |
| **Fam179a** | **family with sequence similarity 179, member A** | **8.81** | **1.15** | **0.45** | **2.55** | **0.011** | **1.000** |
| **Sema6a** | **sema domain, transmembrane domain (TM), and cytoplasmic domain, (semaphorin) 6A** | **13.89** | **1.18** | **0.57** | **2.06** | **0.039** | **1.000** |
| **Naa11** | **N(alpha)-acetyltransferase 11, NatA catalytic subunit** | **6.81** | **1.19** | **0.57** | **2.10** | **0.036** | **1.000** |
| **Tle1** | **transducin-like enhancer of split 1 (E(sp1) homolog, Drosophila)** | **10.88** | **1.20** | **0.42** | **2.88** | **0.004** | **1.000** |
| **Peg3** | **paternally expressed 3** | **10.88** | **1.21** | **0.32** | **3.73** | **<0.001** | **0.456** |
| **Ino80b** | **INO80 complex subunit B** | **28.15** | **1.26** | **0.45** | **2.82** | **0.005** | **1.000** |
| **Fkbp9** | **FK506 binding protein 9** | **12.3** | **1.28** | **0.49** | **2.59** | **0.010** | **1.000** |
| **Xlr4a** | **Protein Xlr4a; Similar to X-linked lymphocyte regulated gene 4 (Predicted)** | **12.91** | **1.3** | **0.58** | **2.23** | **0.026** | **1.000** |
| **Cryaa** | **crystallin, alpha A** | **5.98** | **1.33** | **0.42** | **3.19** | **0.001** | **1.000** |
| **Nfe2l3** | **nuclear factor, erythroid 2-like 3** | **6.83** | **1.36** | **0.57** | **2.40** | **0.016** | **1.000** |
| **Palmd** | **palmdelphin** | **20.45** | **1.44** | **0.40** | **3.62** | **<0.001** | **0.582** |
| **En1** | **engrailed 1** | **4.74** | **1.48** | **0.62** | **2.39** | **0.017** | **1.000** |
| **Cyb5r2** | **cytochrome b5 reductase 2** | **2.93** | **1.48** | **0.69** | **2.15** | **0.032** | **1.000** |
| **Kcnk6** | **potassium channel, two pore domain subfamily K, member 6** | **3.91** | **1.49** | **0.74** | **2.02** | **0.044** | **1.000** |
| **Fam188b** | **family with sequence similarity 188, member B** | **5.66** | **1.51** | **0.64** | **2.36** | **0.018** | **1.000** |
| **Camk2n2** | **calcium/calmodulin-dependent protein kinase II inhibitor 2** | **1.97** | **1.57** | **0.70** | **2.24** | **0.025** | **1.000** |
| **Tnf** | **tumor necrosis factor** | **51.62** | **1.61** | **0.74** | **2.17** | **0.030** | **1.000** |
| **Fbln5** | **fibulin 5** | **3.77** | **1.78** | **0.80** | **2.24** | **0.025** | **1.000** |
| **Capn13** | **calpain 13** | **1.70** | **2.04** | **0.95** | **2.14** | **0.033** | **1.000** |
| **Stox1** | **storkhead box 1** | **6.21** | **2.05** | **0.71** | **2.88** | **0.004** | **1.000** |
| **Hist1h3f** | **histone cluster 1, H3f** | **1.47** | **2.09** | **0.89** | **2.35** | **0.019** | **1.000** |
| **Bglap** | **bone gamma-carboxyglutamate (gla) protein** | **81.98** | **2.12** | **0.84** | **2.53** | **0.011** | **1.000** |
| **Mpdz** | **multiple PDZ domain protein** | **1.40** | **2.12** | **0.95** | **2.24** | **0.025** | **1.000** |
| **Senp5** | **SUMO1/sentrin specific peptidase 5** | **4.97** | **2.3** | **0.96** | **2.39** | **0.017** | **1.000** |
| **Usp51** | **ubiquitin specific peptidase 51** | **0.92** | **2.38** | **1.00** | **2.37** | **0.018** | **1.000** |
| **Trh** | **thyrotropin releasing hormone** | **0.75** | **2.43** | **1.09** | **2.24** | **0.025** | **1.000** |
| **Nxf2** | **nuclear RNA export factor 2** | **2.64** | **2.46** | **0.95** | **2.60** | **0.009** | **1.000** |
| **Fxyd6** | **FXYD domain-containing ion transport regulator 6** | **6.06** | **2.52** | **0.78** | **3.25** | **0.001** | **1.000** |
| **Gstm3** | **glutathione S-transferase mu 3** | **0.81** | **2.54** | **1.26** | **2.01** | **0.044** | **1.000** |
| **Fam47e** | **family with sequence similarity 47, member E** | **3.99** | **2.55** | **1.03** | **2.46** | **0.014** | **1.000** |
| **Eppin** | **epididymal peptidase inhibitor** | **0.83** | **2.58** | **1.21** | **2.14** | **0.033** | **1.000** |
| **Usp13** | **ubiquitin specific peptidase 13** | **0.87** | **2.72** | **1.21** | **2.25** | **0.024** | **1.000** |
| **Pax1** | **paired box 1** | **0.91** | **3.12** | **1.30** | **2.40** | **0.016** | **1.000** |
| **Kcnh1** | **potassium channel, voltage gated eag related subfamily H, member 1** | **1.77** | **3.39** | **0.96** | **3.51** | **<0.001** | **0.582** |
| **Ctrb1** | **chymotrypsinogen B1** | **72.1** | **10.47** | **1.94** | **5.40** | **<0.001** | **0.001** |
