# Supplementary material for: Maternal Western diet programs persistent transcriptomic signatures in offspring PBMCs that are largely reversible by interventions during lactation
Source: Front Nutr. 2026 Jul 22;13:1892621. doi: 10.3389/fnut.2026.1892621 (PMC13440078; doi:10.3389/fnut.2026.1892621)
Supplement: Supplementary file 2 [file Table_2.DOCX]

**Supplementary Table 2.** Multi-group comparison of the set of differentially expressed genes (DEGs) across control (O-C), offspring of Western diet dams (O-WD), offspring of Western diet dams with leptin supplementation (O-WD-LEP), and offspring of reversion dams (O-REV). Statistical comparisons were conducted using the Kruskal–Wallis test followed by post-hoc analysis. Different letters denote statistically significant differences between groups (*p* < 0.05). Genes are ordered according to their post-hoc pattern.

| **Gene symbol** | **C-Veh** | **WD-Veh** | **WD-Lep** | **Rev-Veh** | **Kruskal-Wallis test** | **post-hoc** |
| --- | --- | --- | --- | --- | --- | --- |
| *Spag1* | 49.3±3.5 | 78.3±6.5 | 38.9±7.5 | 38.8±5.9 | p=0.001 | A-B-A-A |
| *Hes5* | 65.2±6.6 | 106.8±9 | 53.4±7.1 | 57.6±8.7 | p=0.001 | A-B-A-A |
| *Ticam2* | 87.5±10.8 | 131.7±11.4 | 91.9±11.9 | 61.3±7.6 | p=0.002 | A-B-A-A |
| *Scd1* | 446.7±54.7 | 723.3±51.1 | 436.7±72.8 | 368.9±55.4 | p=0.002 | A-B-A-A |
| *Fam179a* | 4.9±1.4 | 12.8±2 | 4.3±1.3 | 4.8±0.9 | p=0.002 | A-B-A-A |
| *Chst3* | 296.3±28.8 | 490.4±62.3 | 283.1±47.5 | 258.5±31.5 | p=0.002 | A-B-A-A |
| *Pcbd1* | 13.4±1.7 | 22±2.3 | 10.2±2 | 11.3±1.5 | p=0.002 | A-B-A-A |
| *Peg3* | 6.3±1.2 | 17±2.6 | 7.7±2 | 6.3±1.7 | p=0.003 | A-B-A-A |
| *Cryaa* | 3.2±0.7 | 9.5±1.9 | 4.6±1.2 | 3.2±1.2 | p=0.003 | A-B-A-A |
| *Traf5* | 441.5±40.2 | 686.3±41.6 | 468.5±61 | 409.2±47.9 | p=0.003 | A-B-A-A |
| *Spib* | 1604±146.6 | 2319.9±102.8 | 1584.1±250.3 | 1403.5±188.8 | p=0.003 | A-B-A-A |
| *Stat2* | 2511.9±192.8 | 3392.6±193.9 | 2396.3±251.2 | 2071.2±230.5 | p=0.003 | A-B-A-A |
| *Klhl14* | 171.7±14.9 | 248.9±17.4 | 158.5±24.2 | 142.8±18.1 | p=0.003 | A-B-A-A |
| *Irak3* | 1265.8±112.7 | 1750.8±102 | 1185.8±161 | 1004.8±126.9 | p=0.003 | A-B-A-A |
| *Lin7b* | 13.8±1.5 | 25.2±2.9 | 12.7±2.1 | 13.2±2.8 | p=0.004 | A-B-A-A |
| *Trh* | 0.1±0.1 | 1.5±0.5 | 0.2±0.2 | 0.8±0.4 | p=0.004 | A-B-A-A |
| *Rslcan18* | 114.4±15.3 | 164.9±12.6 | 98.5±12.4 | 95.6±11.7 | p=0.004 | A-B-A-A |
| *Parp16* | 18.2±1.6 | 33.5±4 | 17.2±2.5 | 19.5±3.2 | p=0.005 | A-B-A-A |
| *Syvn1* | 2364±158.5 | 3288.3±186.4 | 2293.8±270 | 2028.6±257.9 | p=0.005 | A-B-A-A |
| *Tor4a* | 739±62.1 | 1006.2±56.6 | 711.9±97.4 | 588.3±80.2 | p=0.005 | A-B-A-A |
| *Chst1* | 305.6±32.3 | 472.9±33.4 | 315.9±54.2 | 268.7±42.3 | p=0.005 | A-B-A-A |
| *Kctd19* | 3.7±0.7 | 7.8±1 | 3.9±0.8 | 4±0.5 | p=0.005 | A-B-A-A |
| *Srpk3* | 291±27.6 | 469.5±35.3 | 302.4±31.3 | 300.4±44.2 | p=0.005 | A-B-A-A |
| *Fcrl1* | 738.1±81.8 | 1060.5±60.8 | 647.8±78.1 | 668.1±94.9 | p=0.005 | A-B-A-A |
| *Faim3* | 4275.8±533.9 | 6413.1±363.6 | 4578.8±532 | 3898.5±520.6 | p=0.005 | A-B-A-A |
| *Lamc1* | 220±24.8 | 306.8±24.6 | 193.7±33.5 | 182.2±22.5 | p=0.005 | A-B-A-A |
| *Swap70* | 2092.6±187.4 | 3101.1±209 | 2108.2±316.4 | 1843.2±258.7 | p=0.006 | A-B-A-A |
| *Myo1e* | 327.7±40.6 | 485.5±42.3 | 316.5±56.6 | 266.5±33.8 | p=0.007 | A-B-A-A |
| *Ache* | 213±22.4 | 334.8±31.5 | 198.7±31.3 | 185.8±22.2 | p=0.008 | A-B-A-A |
| *Caskin2* | 32.7±5.3 | 50.6±3.8 | 32.3±5.4 | 25.2±4.4 | p=0.008 | A-B-A-A |
| *Blk* | 548.5±55.7 | 759.3±45.5 | 524.6±74.1 | 466.1±61.4 | p=0.008 | A-B-A-A |
| *P4ha3* | 9.1±1.7 | 16.2±1.8 | 7.7±1.2 | 8.8±1.6 | p=0.009 | A-B-A-A |
| *Smad1* | 174.5±19.3 | 242.5±15.5 | 156.2±23.5 | 152.8±14.4 | p=0.009 | A-B-A-A |
| *Khdrbs3* | 49.2±7.1 | 99.2±14.2 | 44.8±11.1 | 54.7±9.6 | p=0.011 | A-B-A-A |
| *Usp51* | 0.2±0.1 | 1.6±0.4 | 0.4±0.3 | 0.5±0.3 | p=0.011 | A-B-A-A |
| *Mcm3* | 1923.1±133.6 | 2550±153.6 | 1943±233.1 | 1613.7±187.2 | p=0.011 | A-B-A-A |
| *Lzts1* | 43.2±7.4 | 73.8±7 | 53.1±10 | 36±6.3 | p=0.012 | A-B-A-A |
| *Mtfmt* | 95.3±8.9 | 133.5±9.2 | 96±10.9 | 85.7±10.4 | p=0.014 | A-B-A-A |
| *Fcer2* | 2074.4±171.2 | 3065.3±200.7 | 1959.8±284.8 | 1867.3±253.6 | p=0.014 | A-B-A-A |
| *Tnfrsf13c* | 200.4±29.2 | 328.1±16.8 | 243.3±39.4 | 204.3±32 | p=0.017 | A-B-A-A |
| *Spon1* | 27.3±3.1 | 39.8±3.4 | 29.7±3 | 23.8±3.8 | p=0.017 | A-B-A-A |
| *Camk2a* | 56.6±8.6 | 115.9±17.9 | 65.2±9.6 | 69±12 | p=0.023 | A-B-A-A |
| *Sox12* | 86.4±5.8 | 133.5±12.9 | 87.6±14.9 | 76.6±11.3 | p=0.023 | A-B-A-A |
| *Ctf1* | 18.9±2.2 | 31.5±4 | 19.9±4 | 15.7±2.7 | p=0.025 | A-B-A-A |
| *Slfnl1* | 22.9±2.5 | 36.3±2.9 | 27.4±4.3 | 24.3±3 | p=0.031 | A-B-A-A |
| *Pcdhb19* | 3.3±0.9 | 7.8±1.9 | 2.9±0.6 | 3.9±1.7 | p=0.034 | A-B-A-A |
| *Homez* | 20.4±2.6 | 33.5±3.4 | 22.4±2.5 | 20.2±4.6 | p=0.036 | A-B-A-A |
| *Tnfsf15* | 2.2±0.6 | 0.7±0.3 | 0.5±0.3 | 0.9±0.4 | p=0.040 | A-B-A-A |
| *Lsr* | 99.5±6.5 | 148.2±13.8 | 109.3±13 | 101.4±12.5 | p=0.049 | A-B-A-A |
| *En1* | 2.2±0.5 | 7.5±2.4 | 2.3±0.6 | 3.8±1.3 | p=0.047 | A-B-A-AB |
| *Rmnd5b* | 46.3±10.4 | 15.9±4.2 | 38.1±7.5 | 40.7±15.2 | p=0.049 | A-B-A-AB |
| *Sema6a* | 8.4±3 | 20.2±4.5 | 8.6±2 | 3.4±1.5 | p=0.003 | A-B-AB-A |
| *Pax1* | 0±0 | 1.9±0.8 | 0.8±0.4 | 0.3±0.3 | p=0.006 | A-B-AB-A |
| *Gstm3* | 0.1±0.1 | 1.4±0.5 | 0.4±0.2 | 0.1±0.1 | p=0.009 | A-B-AB-A |
| *Kcnh1* | 0.2±0.1 | 3.1±0.8 | 2±0.9 | 1±0.4 | p=0.013 | A-B-AB-A |
| *Creb3l1* | 9.8±1.6 | 17.9±2.1 | 12.7±2.3 | 9.3±1.9 | p=0.019 | A-B-AB-A |
| *Krt15* | 8.8±3.8 | 0.1±0.1 | 5.4±2.6 | 5.4±2.3 | p=0.020 | A-B-AB-A |
| *Hist1h3f* | 0.5±0.3 | 2.6±0.7 | 1.5±0.6 | 0.7±0.3 | p=0.022 | A-B-AB-A |
| *Fxyd6* | 1.5±0.3 | 8.9±3.2 | 13.3±3.2 | 11.8±7.2 | p=0.023 | A-B-AB-A |
| *Id3* | 227.2±25.6 | 359±32.9 | 301.8±39.9 | 234.4±41.3 | p=0.024 | A-B-AB-A |
| *Acat2* | 98.6±13.6 | 172.2±22.2 | 125.9±21.2 | 93.2±15.1 | p=0.026 | A-B-AB-A |
| *Sbk1* | 18.3±3.7 | 35.3±7.5 | 22.7±2.8 | 16.3±3 | p=0.031 | A-B-AB-A |
| *Dok4* | 94.9±19.5 | 157.5±20.9 | 167.6±30.4 | 88.3±10.4 | p=0.033 | A-B-AB-A |
| *Kif23* | 98.6±11.5 | 163.4±18.8 | 124.3±26.6 | 94.7±18.3 | p=0.043 | A-B-AB-A |
| *Prr36* | 22.3±3.2 | 43.4±7.3 | 24.3±3.8 | 21.5±5 | p=0.045 | A-B-AB-A |
| *Cdkn1c* | 31.3±3.8 | 45.9±3.7 | 33.6±4.4 | 28.3±3.7 | p=0.045 | A-B-AB-A |
| *Hhex* | 436.9±38.2 | 635.7±50.3 | 524.3±60.6 | 435.3±56.3 | p=0.045 | A-B-AB-A |
| *Vps37c* | 35.8±9.1 | 79.7±13.9 | 44.7±8.8 | 40.4±7.7 | p=0.048 | A-B-AB-A |
| *Palmd* | 10.2±1.6 | 31.3±4.4 | 27.5±8.9 | 10.1±2.1 | p=0.002 | AB-C-AC-B |
| *Fam47e* | 1.2±0.8 | 7.3±2.3 | 5±1.6 | 0.4±0.3 | p=0.008 | AB-C-AC-B |
| *Zdhhc2* | 54.7±6.2 | 91.5±9.2 | 83.7±15.5 | 45.7±8.7 | p=0.009 | AB-C-AC-B |
| *Zfp467* | 450.1±40.6 | 637±56.7 | 494.9±63.7 | 385.8±43.9 | p=0.022 | A-B-AB-A |
| *Vwa5a* | 1.9±0.7 | 0±0 | 0.8±0.4 | 0.5±0.3 | p=0.024 | A-B-AB-AB |
| *Edn1* | 29.5±10.3 | 8.5±2.1 | 5.8±3 | 5.6±1.3 | p=0.034 | A-B-AB-B |
| *Prss8* | 7.4±1.4 | 3.3±0.7 | 4.3±1 | 3.1±0.7 | p=0.039 | A-B-AB-B |
| *Enah* | 7.8±2.1 | 3.8±0.7 | 3.3±1 | 2.7±0.4 | p=0.045 | A-B-AB-B |
| *Plet1* | 3.2±0.9 | 0.3±0.2 | 0.7±0.5 | 1.8±0.9 | p=0.010 | A-B-B-AB |
| *Tle1* | 6.3±1.2 | 16.5±3.9 | 17.3±4.8 | 11±2 | p=0.024 | A-B-B-AB |
| *N4bp3* | 11.2±3.2 | 3.7±1.5 | 5.7±4.1 | 5.8±1.5 | p=0.032 | A-B-B-AB |
| *Scgb1a1* | 13.8±8.4 | 0.3±0.2 | 6.3±4.8 | 7.9±4.1 | p=0.036 | A-B-B-AB |
| *Bglap* | 31.4±14.2 | 138.5±39.7 | 203.2±62.9 | 112.4±64.1 | p=0.031 | A-BC-B-AC |
| *Olr59* | 4.2±0.9 | 10.1±2.1 | 11.8±3.1 | 4.8±1.8 | p=0.004 | A-B-B-A |
| *Fkbp9* | 7.1±2.3 | 17.3±2.2 | 16.1±3.2 | 9.8±4.2 | p=0.009 | A-B-B-A |
| *Snta1* | 19.9±2.1 | 46.1±9.2 | 39.8±5.9 | 22.6±4.7 | p=0.017 | A-B-B-A |
| *Ifi27* | 3100.2±729 | 7297.8±1149 | 5698.9±654.6 | 3743.1±827.3 | p=0.014 | A-B-BC-AC |
| *Upk1b* | 1.4±0.5 | 0±0 | 0.7±0.5 | 1.1±0.6 | p=0.019 | A-B-BC-AC |
| *Wnt10b* | 5.6±0.8 | 2.7±0.7 | 2.5±1.2 | 1.5±0.5 | p=0.005 | A-B-B-B |
| *Dpt* | 236.7±44.8 | 106.6±30.1 | 132.1±36.3 | 69.1±12.2 | p=0.007 | A-B-B-B |
| *Ctrb1* | 0.1±0.1 | 126.6±59.3 | 172.4±63.2 | 95±52.6 | p=0.029 | A-B-B-B |
| *Cfap54* | 5.1±1.1 | 2.3±0.8 | 1.4±0.4 | 2.3±0.8 | p=0.042 | A-B-B-B |
| *Casq1* | 82.1±10.7 | 56.2±11.4 | 35.4±8.6 | 38.3±7.3 | p=0.008 | A-AB-B-B |
| *Trib2* | 6581.6±651.8 | 5844.5±729.2 | 4064.6±632.7 | 4107.2±634.1 | p=0.020 | A-AB-B-B |
| *Arid5a* | 3278.3±424.5 | 2734.2±277.4 | 2143±279.9 | 1941.8±204.5 | p=0.027 | A-AB-B-B |
| *Ptprn2* | 2.6±0.7 | 0.7±0.3 | 0.5±0.2 | 0.4±0.2 | p=0.041 | A-AB-B-B |
| *Adamts4* | 5.2±1.7 | 2.2±0.7 | 1.4±0.4 | 1.8±1 | p=0.047 | A-AB-B-B |
| *Tgm1* | 64.7±15 | 45.1±6.3 | 40.6±12.5 | 35.2±6.2 | p=0.047 | A-AB-B-B |
| *Tnf* | 25.9±9.5 | 72.3±35.9 | 13.7±5.2 | 7.2±2.6 | p=0.005 | AB-A-B-B |
| *Mrpl51* | 179.6±16.7 | 237.1±18.4 | 172.8±21.5 | 136.6±17.3 | p=0.005 | AB-A-B-B |
| *Gpha2* | 3.4±1 | 1±0.3 | 4.7±1.4 | 5.2±0.8 | p=0.008 | AB-A-B-B |
| *Arhgap17* | 3818.9±275.8 | 4865.5±321.8 | 3412±447.2 | 2960.3±383.9 | p=0.008 | AB-A-B-B |
| *Papd7* | 788.8±72 | 1042±76.5 | 658.6±98.1 | 598.9±83.5 | p=0.008 | AB-A-B-B |
| *Slc12a4* | 411.8±38.8 | 570.8±43.7 | 398.7±55.7 | 331.9±43.8 | p=0.010 | AB-A-B-B |
| *Ms4a1* | 1281.3±135.5 | 1822.5±142 | 1284±212.8 | 1043.7±121.2 | p=0.010 | AB-A-B-B |
| *Tmc8* | 3772.2±279.5 | 4979.4±350 | 3479.6±468.1 | 3101.8±394.8 | p=0.011 | AB-A-B-B |
| *Acsl3* | 200.3±18.8 | 284±27 | 174.6±28 | 150.8±21.8 | p=0.011 | AB-A-B-B |
| *Sqle* | 240±23.8 | 321.5±21.3 | 211.2±27.9 | 201.6±27.2 | p=0.013 | AB-A-B-B |
| *Slc11a2* | 479±33.4 | 606.5±36.3 | 465.2±51.5 | 393.8±47.3 | p=0.014 | AB-A-B-B |
| *Gba2* | 1528.8±118.7 | 1959.8±136.8 | 1444.9±185.7 | 1259.8±145.2 | p=0.015 | AB-A-B-B |
| *Hmgb1* | 52.6±7.1 | 82.3±9.1 | 42.8±9.4 | 42.8±7.4 | p=0.015 | AB-A-B-B |
| *Parp1* | 2802.8±177.5 | 3705±217.2 | 2952.1±341.9 | 2449.8±307.3 | p=0.020 | AB-A-B-B |
| *Dimt1* | 264.3±26.4 | 356.5±26.4 | 262.7±34.8 | 217.4±27.2 | p=0.020 | AB-A-B-B |
| *Dnaaf5* | 199.8±19.8 | 266±21.7 | 182.7±25.1 | 168.6±21.1 | p=0.024 | AB-A-B-B |
| *Ttc37* | 1071.4±99.1 | 1434.5±121.4 | 1074.6±162.8 | 842.8±110.6 | p=0.026 | AB-A-B-B |
| *Tial1* | 1663.1±128.9 | 2109.1±153.6 | 1616.9±198.3 | 1347.7±164.3 | p=0.029 | AB-A-B-B |
| *Med18* | 415.2±31.9 | 576.8±55 | 371.1±54.1 | 341.4±49.3 | p=0.032 | AB-A-B-B |
| *Cr2* | 1607.4±254.6 | 2534.9±365.2 | 1543.6±324.8 | 1349.6±193.8 | p=0.037 | AB-A-B-B |
| *Ezr* | 14998.4±1274.3 | 16024.2±1154.9 | 12221±1577.8 | 10204.2±1260.9 | p=0.021 | A-A-AB-B |
| *Hs3st1* | 295.1±45.1 | 244.7±20.9 | 184.8±25.4 | 154.3±23.2 | p=0.023 | A-A-AB-B |
| *Arsi* | 55.1±15.1 | 32.7±2.9 | 33.4±7.7 | 17.8±3.7 | p=0.027 | A-A-AB-B |
| *Socs2* | 557.9±60 | 527.4±47.5 | 410.1±65.7 | 338.8±51.2 | p=0.031 | A-A-AB-B |
| *Hnrnpf* | 10839.3±746.9 | 12007.5±916.1 | 9510.1±1200.4 | 7838.4±1073.3 | p=0.042 | A-A-AB-B |
| *Ltk* | 8.3±2.7 | 3.3±1.1 | 5.1±1.7 | 11.8±3.2 | p=0.032 | AB-A-A-B |
| *Slc13a4* | 55.9±9 | 98±14.4 | 105.9±23.7 | 47.7±10.8 | p=0.045 | AB-A-A-B |
| *Rps6ka5* | 599.1±66.6 | 881.5±72.8 | 654.5±116.8 | 456.7±68.8 | p=0.011 | AB-A-AB-B |
| *Slfn2* | 8587.2±797.9 | 12022.8±1121.6 | 9262.4±1249.1 | 6986.8±946.7 | p=0.018 | AB-A-AB-B |
| *Srsf9* | 560.4±48.4 | 775.3±68.4 | 643.1±78.9 | 481.7±72.6 | p=0.033 | AB-A-AB-B |
| *Naa11* | 3.8±1.1 | 10.5±3 | 5.9±2.3 | 2.8±1.2 | p=0.042 | AB-A-AB-B |
| *Spata6* | 449.4±31.1 | 467.3±32.6 | 338.5±43.7 | 325.3±44.6 | p=0.021 | AB-A-BC-C |
| *Tp53i3* | 85.8±10.3 | 68.9±8.3 | 53.3±8.6 | 43.8±8.4 | p=0.015 | A-AB-BC-C |
| *Vwa9* | 555.7±37.3 | 563.1±53 | 429.8±52.9 | 390.1±55.1 | p=0.025 | A-AB-BC-C |
| *Npas2* | 21.1±4.4 | 13.5±2.3 | 11.3±1.4 | 7.3±2 | p=0.024 | A-AB-AB-B |
| *Pcdhgb6* | 14.9±3.2 | 8.2±1.6 | 5±1.3 | 7.9±1.9 | p=0.042 | A-AB-B-AB |
| *Etnk2* | 9.6±1.6 | 5±1.3 | 4.8±1.8 | 5.5±1.8 | p=0.052 |  |
| *Eppin* | 0.1±0.1 | 1.5±0.5 | 0.6±0.3 | 1±0.4 | p=0.052 |  |
| *Stox1* | 2.3±1.1 | 11.5±5.1 | 21.8±8 | 6.1±3 | p=0.054 |  |
| *Bpifa1* | 13.7±8.4 | 0.2±0.2 | 14.5±9 | 17.7±11.4 | p=0.056 |  |
| *Pagr1* | 321.5±26.2 | 486.3±49.3 | 433.8±56.6 | 322.7±39.3 | p=0.058 |  |
| *A4gnt* | 14.9±1.6 | 25.1±3.7 | 14.1±2.7 | 13.6±3.3 | p=0.058 |  |
| *Sulf1* | 35.2±10.3 | 20.1±2.4 | 15.8±3.2 | 11.8±2.9 | p=0.059 |  |
| *Fbxo39* | 36.4±4.2 | 56.5±7.9 | 38.1±6.3 | 30.8±6.6 | p=0.059 |  |
| *Slc30a1* | 391.7±26.1 | 407.9±31.9 | 339.1±39.4 | 288.2±31.8 | p=0.060 |  |
| *Cd63* | 1486.6±287.8 | 812.5±176.2 | 587.3±84.8 | 968.1±335.3 | p=0.061 |  |
| *Cidec* | 18.6±4.5 | 33.9±5.1 | 19.3±4.5 | 20.3±2.3 | p=0.061 |  |
| *Tonsl* | 47.5±7.2 | 73±6.5 | 48.9±9.6 | 50.3±6.3 | p=0.061 |  |
| *Camk2n2* | 1±0.3 | 3.5±0.9 | 1±0.3 | 1.4±0.5 | p=0.062 |  |
| *Sfrp5* | 8.6±1.7 | 17.8±3.1 | 13.6±2.4 | 9.6±1.8 | p=0.062 |  |
| *Tle3* | 4033.7±340.8 | 4272.6±335.7 | 3294.8±403.9 | 2844.8±392.2 | p=0.064 |  |
| *Cyb5r2* | 1.4±0.4 | 4.3±1.3 | 1.6±0.7 | 2±0.5 | p=0.064 |  |
| *Eya2* | 193.4±30 | 170.8±15 | 142.7±22.9 | 112.8±15.6 | p=0.064 |  |
| *Cd300ld* | 4.3±2.1 | 0.9±0.6 | 1.1±0.5 | 0.4±0.3 | p=0.065 |  |
| *Nxf2* | 5.3±4.5 | 5.3±2.7 | 1.1±0.7 | 1±0.4 | p=0.067 |  |
| *Pgf* | 12.6±3.2 | 23.4±2.7 | 16.3±3.1 | 19.3±4 | p=0.068 |  |
| *Odf3b* | 23.6±2.4 | 43.1±6.8 | 24.3±3.4 | 29.9±5 | p=0.068 |  |
| *Gpx8* | 2.9±0.7 | 1.1±0.4 | 1.2±0.6 | 1.3±0.4 | p=0.069 |  |
| *Atp13a4* | 2.5±1 | 0.5±0.4 | 0.6±0.3 | 0.3±0.2 | p=0.069 |  |
| *Mtmr10* | 289.7±20.7 | 279.4±26.3 | 211.7±34.4 | 210±30 | p=0.070 |  |
| *Lamc3* | 20.6±2.2 | 33.7±4.2 | 24±6.5 | 20.4±2.1 | p=0.070 |  |
| *Ccdc27* | 1.3±0.4 | 0.4±0.3 | 0.8±0.4 | 1.2±0.6 | p=0.070 |  |
| *Ramp2* | 7.5±1.4 | 3.4±0.7 | 3.7±0.8 | 3.7±1 | p=0.071 |  |
| *Coro2b* | 113.5±29 | 66.9±8.6 | 45.8±11 | 48.2±12.6 | p=0.071 |  |
| *Usp13* | 0.1±0.1 | 1.8±0.7 | 1.3±0.6 | 0.9±0.3 | p=0.072 |  |
| *Lama4* | 7.1±1.7 | 14.6±3.4 | 11.3±2.3 | 7.5±1.7 | p=0.072 |  |
| *Thbs3* | 33.4±4.5 | 27.3±4 | 20.5±3.5 | 20.9±3.3 | p=0.074 |  |
| *Cycs* | 111.7±11.7 | 105.8±11.2 | 99±12.9 | 69.7±10.7 | p=0.075 |  |
| *Snip1* | 32.3±10.6 | 20.2±1.8 | 13.2±1.6 | 19.8±4.8 | p=0.076 |  |
| *Ccno* | 3.8±1.6 | 0.2±0.1 | 0.5±0.3 | 0.6±0.2 | p=0.077 |  |
| *Prrt3* | 17.3±2.9 | 13.8±1.2 | 10±0.9 | 12.8±2.1 | p=0.077 |  |
| *Cyp2f4* | 2.8±1 | 0.6±0.5 | 1.2±0.7 | 1.9±0.6 | p=0.079 |  |
| *Pla2g2c* | 4.9±1 | 2.2±0.6 | 2±0.5 | 2.4±0.8 | p=0.081 |  |
| *Cd276* | 10.3±1.1 | 20.1±3.8 | 16.9±2.7 | 10.6±2.4 | p=0.081 |  |
| *Olr288* | 1.9±0.8 | 0.5±0.2 | 0.8±0.4 | 0.3±0.3 | p=0.086 |  |
| *Zfp111* | 4.7±0.9 | 1.8±0.4 | 3.8±1.1 | 2.4±0.8 | p=0.089 |  |
| *Rgs1* | 11287±1747 | 8764.4±1595 | 6568±1330.5 | 6272.3±1178.5 | p=0.090 |  |
| *Asb2* | 17.8±3 | 32.3±5.5 | 25.8±4.1 | 18.8±5.5 | p=0.093 |  |
| *Wbp11* | 36.2±16.4 | 10±2.4 | 9±3.3 | 27.5±19.8 | p=0.096 |  |
| *Zfp260* | 1.7±0.5 | 0.4±0.2 | 0.8±0.3 | 0.3±0.2 | p=0.101 |  |
| *Mpdz* | 0.5±0.3 | 2.7±0.8 | 1.6±0.6 | 1.1±0.4 | p=0.103 |  |
| *Kcnk6* | 2±0.6 | 5.9±2 | 7.1±3.3 | 1.7±0.7 | p=0.103 |  |
| *Zfp605* | 19.6±2.2 | 17±2 | 13±1.5 | 13.8±2.9 | p=0.105 |  |
| *Pla2g4cl1* | 8.5±4.3 | 0.5±0.5 | 3.3±2.3 | 5.8±2.6 | p=0.107 |  |
| *Aqp5* | 2.1±0.8 | 0.3±0.3 | 1±0.5 | 2.6±1.7 | p=0.107 |  |
| *Ifitm1* | 3050.1±298.1 | 2853.6±268.6 | 2598.6±314.2 | 2077.5±235.5 | p=0.108 |  |
| *Gli1* | 19±2.3 | 13.7±1.6 | 14.8±2.7 | 10.9±1.7 | p=0.109 |  |
| *Slc52a3* | 22.5±3.8 | 37.6±4.4 | 29.3±4.2 | 26.5±3.5 | p=0.109 |  |
| *Kif22* | 68.4±9.8 | 100.6±9.7 | 90.2±17.4 | 74.7±13.6 | p=0.111 |  |
| *Tac1* | 6.6±1.8 | 1.7±0.5 | 5.7±1.6 | 4.3±1.4 | p=0.113 |  |
| *F12* | 23.7±6.2 | 12.1±4.3 | 9.6±2.9 | 10.7±3.3 | p=0.114 |  |
| *Plcb4* | 224.6±36.6 | 194.5±19.8 | 176.6±26.7 | 129.4±15.3 | p=0.116 |  |
| *Ctla4* | 826.6±85.4 | 728.7±86.8 | 607.3±83 | 564.1±100.3 | p=0.121 |  |
| *Prrt2* | 47.7±5.6 | 81.5±11.2 | 56.8±10.5 | 50.2±9.5 | p=0.122 |  |
| *Rassf6* | 16.2±1.7 | 11.2±1.9 | 11.1±2 | 13.9±1.7 | p=0.127 |  |
| *Plekhf1* | 62.8±13.7 | 39.5±4.6 | 39.8±7.3 | 27.6±4.4 | p=0.127 |  |
| *Lipt2* | 38.6±4.4 | 59.8±6.6 | 45.4±6.7 | 40.7±6.2 | p=0.130 |  |
| *Clca1* | 16.1±6.5 | 0.5±0.2 | 9.1±4.7 | 12.2±5.1 | p=0.131 |  |
| *Cyp26b1* | 3.6±0.8 | 1.8±0.6 | 3.5±1 | 1.6±0.6 | p=0.131 |  |
| *Chchd2* | 1.2±0.4 | 0.2±0.1 | 0.6±0.3 | 0.8±0.6 | p=0.132 |  |
| *Rln1* | 6.6±2 | 1.9±0.5 | 2±0.9 | 1.4±0.6 | p=0.135 |  |
| *Adrbk2* | 122.2±19.1 | 105.5±8.9 | 84.3±13.6 | 75.2±11.3 | p=0.137 |  |
| *Ankrd29* | 3.2±0.7 | 1.5±0.5 | 2.2±0.6 | 1.4±0.5 | p=0.140 |  |
| *Xlr4a* | 6.8±1.4 | 22.6±9.7 | 5.4±2.2 | 5.8±1.9 | p=0.144 |  |
| *Nbl1* | 16.8±3 | 29.8±4.6 | 23.6±4.6 | 19.5±3.7 | p=0.144 |  |
| *Ints1* | 14.8±8.5 | 1.5±0.5 | 6.3±5 | 1.2±0.4 | p=0.145 |  |
| *Rasal2* | 14.6±3.2 | 6.6±1.6 | 7.5±2.3 | 5.4±1.3 | p=0.150 |  |
| *Xk* | 298.1±40 | 578.2±88.2 | 469.2±121.3 | 337.6±61.7 | p=0.153 |  |
| *Cacng4* | 132.4±41.8 | 77.2±9 | 73.3±18.5 | 50.3±8 | p=0.153 |  |
| *Krt13* | 2.2±1.3 | 0±0 | 1.7±0.8 | 0.8±0.5 | p=0.154 |  |
| *Krt23* | 1.9±0.9 | 0.2±0.2 | 2.6±1 | 2.2±1 | p=0.155 |  |
| *Pask* | 41.8±5.7 | 63.6±7.6 | 43.8±6.8 | 43.9±8.3 | p=0.157 |  |
| *Fbln1* | 417.7±62.8 | 268.5±58.8 | 470.9±57.3 | 463.7±129.3 | p=0.158 |  |
| *Hist1h2ah* | 13.2±2.8 | 26.9±5.1 | 21.8±4.3 | 14.1±2.4 | p=0.162 |  |
| *Als2cr12* | 33.2±4.6 | 26.7±3.2 | 28.6±3 | 22.3±2.5 | p=0.163 |  |
| *Rpl7a* | 1192.5±145.7 | 2181.5±538.3 | 1421.8±235.3 | 2114.1±1124.8 | p=0.164 |  |
| *Bcl2* | 1272.6±212.3 | 1024.6±138.8 | 786.6±114.3 | 747.2±85.3 | p=0.167 |  |
| *Nsun4* | 8.5±3.7 | 2.4±0.6 | 2.6±0.6 | 2.3±1.2 | p=0.169 |  |
| *Nfe2l3* | 3.7±0.8 | 10.4±3 | 4.8±1.5 | 3.6±1.1 | p=0.172 |  |
| *Slc27a2* | 271.6±98.2 | 94.5±36.2 | 162.6±60.8 | 199±50.7 | p=0.176 |  |
| *Apba1* | 72.3±11.4 | 55.6±8.6 | 60.5±10.7 | 39.4±7.7 | p=0.179 |  |
| *Nsun4* | 8.6±3.2 | 4.5±0.9 | 3.4±0.6 | 4.2±1.1 | p=0.185 |  |
| *Rab7b* | 157±16.2 | 151.6±15.3 | 143.8±20.5 | 107.7±14.1 | p=0.188 |  |
| *Slc39a4* | 1.4±0.5 | 0.2±0.1 | 2±0.9 | 2.1±0.9 | p=0.191 |  |
| *Fhl2* | 258±22.1 | 235.6±33.4 | 216.7±41.7 | 170.4±30.2 | p=0.192 |  |
| *Nbn* | 354.4±38.8 | 296.6±45 | 315.9±53.5 | 241±33.6 | p=0.194 |  |
| *Art2b* | 2817.9±375.9 | 2516.5±327.9 | 1823.7±295.8 | 2123.6±294.4 | p=0.197 |  |
| *Epdr1* | 43±5.3 | 77.8±13.8 | 60.3±13.2 | 40.8±7.5 | p=0.198 |  |
| *Ak9* | 17.1±2.2 | 35.8±8 | 22.2±3.5 | 18.4±3.1 | p=0.199 |  |
| *Col2a1* | 6.5±3.6 | 2.4±0.8 | 1.3±0.5 | 3.1±1.4 | p=0.203 |  |
| *Myo1b* | 9.3±2.7 | 2.6±0.9 | 3.8±1.2 | 4.7±1.6 | p=0.208 |  |
| *Fam188b* | 2.7±0.9 | 8.5±2 | 6.4±1.8 | 6.6±2 | p=0.208 |  |
| *Olfml3* | 21.3±2.4 | 34.3±4.4 | 28.3±5.7 | 24.4±3.2 | p=0.208 |  |
| *Eef2k* | 1086.7±80.7 | 1110.7±68.9 | 987.6±87.2 | 903.1±73.2 | p=0.213 |  |
| *Rpl39* | 40.4±4.5 | 35.5±4.2 | 41.8±5.2 | 29.4±4.2 | p=0.215 |  |
| *Igf1* | 4.1±1.5 | 1.4±0.3 | 1.3±0.6 | 3.1±1.1 | p=0.218 |  |
| *Ewsr1* | 161.5±21.5 | 260.6±53.7 | 154.3±23.8 | 214.4±41.2 | p=0.229 |  |
| *Rgs8* | 4.9±1.7 | 1.3±0.4 | 2.9±0.9 | 2.1±0.9 | p=0.231 |  |
| *Kcnma1* | 3.4±0.9 | 1.3±0.5 | 2.3±0.5 | 2.1±0.8 | p=0.239 |  |
| *Socs1* | 1390±171 | 1184.3±185.9 | 1150.8±178.4 | 910.3±186 | p=0.243 |  |
| *P2ry2* | 156.9±19.6 | 139.1±14.2 | 126.8±23 | 101.4±18.8 | p=0.256 |  |
| *Ebna1bp2* | 135.3±48.4 | 51.9±19.1 | 106.8±62.9 | 76.1±32.9 | p=0.256 |  |
| *Dfna5* | 26.8±3.9 | 21.7±2.5 | 18.4±3 | 17.8±2.3 | p=0.261 |  |
| *Capn13* | 0.6±0.3 | 3.1±1.3 | 1.6±0.6 | 1.2±0.4 | p=0.264 |  |
| *Slc26a10* | 53.7±5.8 | 47.2±3.8 | 43.7±6.7 | 38.8±5.4 | p=0.277 |  |
| *Lepr* | 5.1±0.8 | 2.7±0.8 | 3.3±0.8 | 3.3±1 | p=0.277 |  |
| *Zfat* | 481.5±87.2 | 383.9±51.1 | 305.4±54.1 | 415.2±74.7 | p=0.281 |  |
| *Irf6* | 438.9±36.5 | 387.6±59.8 | 394.1±55 | 337.3±72.7 | p=0.288 |  |
| *Slc39a13* | 217.3±18.9 | 210±17.5 | 185±24.5 | 163.1±20 | p=0.300 |  |
| *Senp5* | 1.6±0.5 | 7.6±3.3 | 1±0.4 | 3.6±1.7 | p=0.301 |  |
| *Mmrn2* | 20.2±3.2 | 14.5±1.5 | 15±2.9 | 13±2.8 | p=0.304 |  |
| *Gtpbp6* | 353.6±189.4 | 295.1±39 | 264.3±44.2 | 250.7±49.6 | p=0.312 |  |
| *Sash1* | 11.1±2.4 | 7.3±1.6 | 7.6±1.8 | 6.4±2.2 | p=0.315 |  |
| *Hdac6* | 19.3±4.1 | 42.4±9.1 | 17.7±4.7 | 24.3±7.4 | p=0.315 |  |
| *Depdc7* | 6.4±1.1 | 4.3±1.1 | 6.6±1.6 | 6.3±1.5 | p=0.325 |  |
| *Mt2A* | 41.7±8 | 24.3±4.2 | 35.4±12.5 | 25.8±5.1 | p=0.330 |  |
| *Tspan1* | 3.1±1 | 1.4±0.5 | 2.3±0.7 | 1.4±0.4 | p=0.331 |  |
| *Prdm1* | 639.8±116.2 | 505.8±53.7 | 427.9±76.2 | 422.8±87.7 | p=0.343 |  |
| *Tmprss6* | 10.5±2 | 6.3±1.3 | 6.7±1.3 | 6.6±1.5 | p=0.348 |  |
| *Ino80b* | 25.9±10.7 | 40.5±12.8 | 45.3±25.1 | 57.5±22.3 | p=0.353 |  |
| *Adap2* | 14.5±2.2 | 9.8±1.9 | 12.4±2.9 | 9.6±2.5 | p=0.358 |  |
| *Rhpn1* | 2.6±1.1 | 0.7±0.4 | 1.6±0.5 | 1.5±0.7 | p=0.365 |  |
| *Gpat2* | 14.2±4 | 7.7±1.6 | 6.1±1.6 | 8±2.5 | p=0.372 |  |
| *Krt4* | 2.9±1.8 | 0.2±0.2 | 3.1±1.7 | 2.3±1.2 | p=0.378 |  |
| *Akr1c3* | 4.2±2.2 | 0.5±0.4 | 1.8±1.1 | 1.8±0.9 | p=0.382 |  |
| *Pc* | 115.5±8.7 | 106.2±8.2 | 109.1±11.4 | 89.8±11.3 | p=0.387 |  |
| *Ndufa1* | 120±81.5 | 74.5±39.6 | 118.1±56.3 | 53.3±41 | p=0.388 |  |
| *Nog* | 3.8±2.4 | 0.3±0.2 | 2.9±2.9 | 1.9±1.3 | p=0.396 |  |
| *Ltc4s* | 14.3±4.1 | 8.6±1.4 | 13.2±2 | 18.2±6.2 | p=0.421 |  |
| *Acacb* | 25.8±5.7 | 16.1±2.4 | 21.8±4.5 | 23.4±3 | p=0.424 |  |
| *Edaradd* | 41.1±13.8 | 19.3±3.9 | 22.4±6.7 | 16.5±3.9 | p=0.429 |  |
| *Cysltr1* | 15.4±5.9 | 7.7±1.8 | 7.1±2.2 | 5.1±1.1 | p=0.431 |  |
| *Txnip* | 32557.6±5332.2 | 27761.6±4205.9 | 28936.3±5535.9 | 22284.7±4128.6 | p=0.441 |  |
| *Cep131* | 818.2±108.6 | 721.4±76.2 | 628.1±65.4 | 703.3±119.2 | p=0.445 |  |
| *Epb41l3* | 6.8±1.1 | 4.5±1 | 6.3±1 | 5.9±1.2 | p=0.446 |  |
| *Triap1* | 16.4±2.9 | 29.8±6.9 | 41.5±16.3 | 22.3±5.5 | p=0.451 |  |
| *Begain* | 379.3±98 | 255±23.5 | 250.8±45.1 | 215.7±26.6 | p=0.452 |  |
| *Golga7b* | 9.2±3 | 4.2±1.5 | 3.3±0.7 | 3.8±0.9 | p=0.463 |  |
| *Hdhd3* | 11.9±1.8 | 8.8±1.5 | 10.1±1.1 | 9.4±2.1 | p=0.467 |  |
| *Scin* | 264.3±109.3 | 136.5±19.4 | 138.4±36.6 | 87.4±16.7 | p=0.479 |  |
| *Dsp* | 16±7.4 | 5.9±1.4 | 8.8±4 | 6.6±2.4 | p=0.490 |  |
| *Gsdma* | 15.5±3.3 | 10.7±1.9 | 11±1.7 | 10.1±1.9 | p=0.492 |  |
| *Fbln5* | 1.6±0.5 | 5.9±2.2 | 6±3.8 | 3.2±1.3 | p=0.493 |  |
| *Etv1* | 16.4±3.1 | 10.5±1.5 | 12.5±3 | 11.1±2.3 | p=0.509 |  |
| *Duox2* | 9.6±2.3 | 5.1±0.8 | 5.6±1.1 | 5.7±1.2 | p=0.532 |  |
| *Oplah* | 357.4±92.7 | 240.9±26.5 | 259.9±40.6 | 206.8±36 | p=0.548 |  |
| *Cndp1* | 13.3±2.6 | 8.4±1.4 | 9.3±2.8 | 9.7±2.4 | p=0.549 |  |
| *Vcan* | 1173.5±253.8 | 915.5±149 | 902.7±153.6 | 692±120 | p=0.559 |  |
| *Spag4* | 5±2.3 | 2±0.5 | 2.2±0.9 | 1.7±0.7 | p=0.573 |  |
| *Bhlha15* | 32.4±5.6 | 25.3±2.4 | 23±3.4 | 34.8±13.8 | p=0.601 |  |
| *Slc9a3* | 1.9±0.9 | 0.4±0.2 | 0.9±0.4 | 0.4±0.2 | p=0.601 |  |
| *Myh1* | 12.8±3.5 | 7.2±1.1 | 7.3±1.6 | 7.8±1.9 | p=0.602 |  |
| *Ltbp2* | 7.5±2.3 | 3.4±0.9 | 3.5±1.1 | 4.3±1.7 | p=0.620 |  |
| *She* | 227.8±39.2 | 196.2±15.2 | 183.7±24.4 | 178.1±18.6 | p=0.640 |  |
| *Krt8* | 10.6±2.7 | 6.5±1.6 | 9.2±2.1 | 8.8±2.9 | p=0.657 |  |
| *Mmel1* | 32.3±7.6 | 19.5±3.7 | 20.8±3.4 | 19.8±1.9 | p=0.683 |  |
| *Slc36a3* | 25.5±2.9 | 21.7±2.8 | 23.2±4.6 | 19.8±3.1 | p=0.726 |  |
| *Fam71b* | 41.7±5.4 | 35.5±3.9 | 35.8±5.2 | 36.2±5.9 | p=0.737 |  |
| *Ighg* | 10±3.8 | 48.1±44 | 10.3±5.9 | 11.5±5.5 | p=0.751 |  |
| *Wfdc2* | 29.3±4.8 | 23.1±3.1 | 29.3±4.3 | 25.9±5.1 | p=0.832 |  |
| *Tbcb* | 104.9±48.6 | 25±10.5 | 15±2.1 | 41.4±21.3 | p=0.928 |  |
| *Cacnb4* | 27.9±12.9 | 8±2.2 | 9.3±3 | 8.8±2.5 | p=0.973 |  |
