# Supplementary material for: Maternal Western diet programs persistent transcriptomic signatures in offspring PBMCs that are largely reversible by interventions during lactation
Source: Front Nutr. 2026 Jul 22;13:1892621. doi: 10.3389/fnut.2026.1892621 (PMC13440078; doi:10.3389/fnut.2026.1892621)
Supplement: Supplementary file 3 [file Table_3.DOCX]

**Supplementary Table 3.** Loadings of necroptosis and NF-κB pathway genes on PCA and PLS-DA components.

|  | Loading PCA | | Loading PLS-DA | |
| --- | --- | --- | --- | --- |
|  | PC1 (31.8%) | PC2  (9.8%) | PC1  (11%) | PC2 (27.8%) |
| *Bcl2* | 0.049547 | 0.066311 | -0.051604 | -0.083906 |
| *Camk2a* | 0.068059 | -0.062957 | 0.08566 | -0.091732 |
| *Edaradd* | 0.043432 | 0.11088 | -0.077786 | -0.064097 |
| *Hist1h2ah* | 0.053924 | -0.051377 | 0.097587 | -0.049544 |
| *Hmgb1* | 0.085067 | -0.019475 | 0.057993 | -0.11513 |
| *Stat2* | 0.092906 | -0.036379 | 0.080756 | -0.12237 |
| *Tnf* | 0.01549 | -0.026653 | 0.035735 | -0.017311 |
| *Tnfrsf13c* | 0.069263 | -0.074912 | 0.10846 | -0.084402 |
| *Traf5* | 0.083395 | -0.071932 | 0.11093 | -0.10527 |
| *Ticam2* | 0.090232 | -0.031448 | 0.082215 | -0.11199 |
| *Parp1* | 0.095552 | -0.037369 | 0.087796 | -0.12114 |
